# Targeted Mutagenesis of the Rice *FW 2.2*-Like Gene Family Using the CRISPR/Cas9 System Reveals OsFWL4 as a Regulator of Tiller Number and Plant Yield in Rice

**DOI:** 10.3390/ijms21030809

**Published:** 2020-01-26

**Authors:** Qingsong Gao, Gang Li, Hui Sun, Ming Xu, Huanhuan Wang, Jianhui Ji, Di Wang, Caiyong Yuan, Xiangxiang Zhao

**Affiliations:** 1 Jiangsu Collaborative Innovation Center of Regional Modern Agriculture & Environmental Protection/Jiangsu Key Laboratory for Eco-Agricultural Biotechnology around Hongze Lake, Huaiyin Normal University, Huai’an 223300, China; 2 Huaiyin Institute of Agricultural Sciences of Xuhuai Region in Jiangsu, Huai’an 223001, China

**Keywords:** *FW2.2*-like gene, tiller number, grain yield, rice, CRISPR/Cas9, genome editing, off-target effect

## Abstract

The *FW2.2*-like (*FWL*) genes encode cysteine-rich proteins with a placenta-specific 8 domain. They play roles in cell division and organ size control, response to rhizobium infection, and metal ion homeostasis in plants. Here, we target eight rice *FWL* genes using the CRISPR/Cas9 system delivered by *Agrobacterium*-mediated transformation. We successfully generate transgenic T_0_ lines for 15 of the 16 targets. The targeted mutations are detected in the T_0_ lines of all 15 targets and the average mutation rate is found to be 81.6%. Transfer DNA (T-DNA) truncation is a major reason for the failure of mutagenesis in T_0_ plants. T-DNA segregation analysis reveals that the T-DNA inserts in transgenic plants can be easily eliminated in the T_1_ generation. Of the 30 putative off-target sites examined, unintended mutations are detected in 13 sites. Phenotypic analysis reveals that tiller number and plant yield of *OsFWL4* gene mutants are significantly greater than those of the wild type. Flag leaves of *OsFWL4* gene mutants are wider than those of the wild type. The increase in leaf width of the mutants is caused by an increase in cell number. Additionally, grain length of *OsFWL1* gene mutants is higher than that of the wild type. Our results suggest that transgene-free rice plants with targeted mutations can be produced in the T_1_ generation using the *Agrobacterium*-mediated CRISPR/Cas9 system and that the *OsFWL4* gene is a negative regulator of tiller number and plant yield.

## 1. Introduction

*fw 2.2* is a major quantitative trait locus that regulates fruit size and weight in tomato [1,2]. The underlying gene *FW2.2* regulates fruit size by negatively regulating cell division [2,3]. Homolog identification and sequence analysis have revealed that FW2.2 belongs to a large eukaryotic family of cysteine-rich proteins containing a featured placenta-specific 8 domain [4,5]. *FW2.2*-like (*FWL*) genes have been characterized in various plant species and are reported to play important roles in diverse biological processes, such as cell number and organ size control [4,6,7,8,9], nodulation [5,10], and metal ion homeostasis [11,12,13,14,15,16,17,18,19,20]. The rice *FWL* gene family contains eight members [9]. Among them, the *OsFWL3* gene is reported to negatively affect grain length and weight by regulating cell division in the glume [9]. However, the *OsFWL4* gene has been reported to affect cadmium (Cd) resistance upon expression in yeast [15]. RNA interference-mediated knockdown of *OsFWL4* has been found to reduce translocation of Cd from the roots to shoots in rice seedlings. More recently, a change in the expression of *OsFWL1* and *OsFWL2* induced by their overexpression or RNA interference has been found to affect Cd tolerance and accumulation in rice [20]. Interestingly, the *OsFWL5*/*PCR1* gene, which affects Cd and Zn tolerance when expressed in yeast cells, has been reported to regulate metal ion homeostasis and grain size and weight in rice [13,14]. However, whether other Cd-responsive rice *FWL* genes also play a role in plant and organ development in rice remains unknown.

Generating mutants with intended mutations is crucial for functional analysis of plant genes. The clustered regularly interspaced short palindromic repeats (CRISPR)/CRISPR-associated protein 9 (Cas9) system is a powerful tool for genome editing in various organisms, including plants. This system induces DNA double-strand breaks at given genomic sites, which are subsequently repaired by either non-homologous end joining or homologous recombination pathways in the cells [21]. Non-homologous end joining is error-prone and can act throughout the cell cycle. It is therefore commonly utilized to disrupt genes by creating random insertions or deletions (indels) at target sites [22]. In the presence of a homologous DNA template, a double-strand break can be repaired by homologous recombination, leading to target gene replacement or insertion. The precise cleavage of the target DNA using the CRISPR/Cas9 system requires two components, namely, Cas9 nuclease harboring HNH and RuvC endonuclease domains for cleaving and an engineered single-guide RNA (sgRNA) for directing Cas9 to the target site [23]. A prerequisite for binding and cleavage of the target DNA is the presence of a trinucleotide protospacer adjacent motif (PAM) immediately after the target DNA [24]. Sequence specificity can be achieved by changing a 20-nucleotide “guide sequence” in the sgRNA. As this system does not require protein engineering, the nuclease can be easily reprogrammed. With the development of highly efficient CRISPR/Cas9 systems, stable homozygous mutants can be obtained within a single generation in many plants [25,26,27,28].

*Agrobacterium*-mediated transfer DNA (T-DNA) transformation is commonly used for delivering CRISPR/Cas9 DNA into rice cells. In this study, two target sites are designed for each of the eight rice *FWL* genes for gene editing using the *Agrobacterium*-mediated CRISPR/Cas9 system. We generate transgenic T_0_ lines from 15 out of 16 constructs and detect targeted mutations in all T_0_ lines. Gene editing efficiency, T-DNA segregation patterns, and off-target effects are analyzed. The phenotypes of homozygous and transgene-free mutants with no detected off-target mutations of the *OsFWL1* and *OsFWL4* genes are then examined. Our results suggest that OsFWL4 is a negative regulator of tiller number and plant yield in rice and that OsFWL1 plays a role in modulating rice grain length.

## 2. Results

### 2.1. Generation of Rice FWL Gene Mutants Using CRISPR/Cas9

Two target sites were designed in the coding region of each of the eight rice *FWL* genes for CRISPR/Cas9 gene editing (Table 1). The GC content in these target sites was in the range 45–75%. The synthesized oligos were inserted into the CRISPR/Cas9 binary vector (Figure S1). Subsequently, the 16 constructed vectors were transformed into the *Japonica* rice variety Zhonghua 11 using the *Agrobacterium*-mediated method.

Of the 16 vectors, we successfully generated transgenic T_0_ lines for 15 vectors (Table 2). We detected targeted mutations in all those T_0_ lines. The mutation rates varied from 26.7% to 100%, and the average mutation rate was 81.6% (Table 2), suggesting that the CRISPR/Cas9 system constructed in this study is efficient in rice gene editing. Bi-allelic mutants were detected in T_0_ plants from each vector, with detection percentages varying from 20.0% to 87.5% (Table 2). Homozygous mutants were detected in T_0_ plants from 13 vectors, with the highest detection percentage being 64.3%. By contrast, heterozygotes and chimeras were detected only in T_0_ plants from three vectors (Table 2). The percentage of heterozygotes and chimeras in all T_0_ plants was only 1.3% and 4.0%, respectively. Detailed sequencing results of all T_0_ mutants are shown in Table S1.

**Figure 1:**
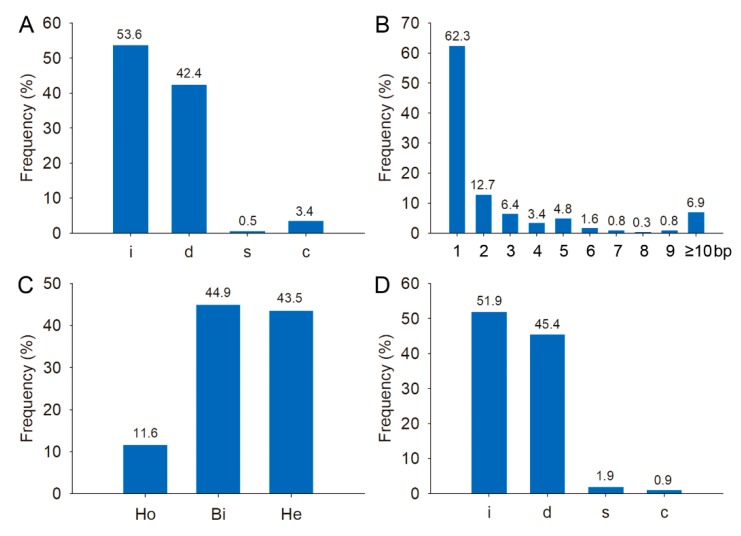
Characterization of on-target and off-target mutations. (**A**) Frequencies of different types of on-target mutations. (**B**) Frequencies of different lengths of on-target mutations. (**C**) Zygosity of off-target mutations. (**D**) Types of off-target mutations. Legend: i, insertion; d, deletion; s, substitution; c, combined mutation; Ho, homozygote; Bi, bi-allele; He, heterozygote.

Sequencing analyses revealed that most mutations were short indels; 62.3% of indels were 1 bp changes (Figure 1A,B). A majority of the 1 bp insertions (83.2%) were either A or T, which is consistent with previous reports [27,29].

Of the 223 T_0_ plants, 41 plants did not contain mutations. To test whether failed editing of these plants was caused by a lack of the CRISPR/Cas9 construct, the presence of *hygromycin phosphotransferase* (*HPT*), sgRNA, and *Cas9* transgenes in these 41 plants was examined. Two plants did not contain *HPT*, sgRNA, and *Cas9* sequences (Table S2), which suggests that these plants escaped hygromycin selection. Twenty-five plants did not contain sgRNA and/or *Cas9* sequences (Table S2) which suggests that incompleteness of the sgRNA/*Cas9* expression cassette led to failed mutagenesis in these plants. Interestingly, when unmutated T_0_ plants without the complete sgRNA/*Cas9* construct were excluded, all targets except Osfwl1a, Osfwl4a, and Osfwl6a had a mutation rate of 100% (Table 2 and Table S2). The score of sgRNA activity in all targets predicted using the sgRNA Scorer 2.0 varied from −0.64 to 1.09 (Table S3), indicating moderate efficiency of the sgRNAs [30].

The inheritance patterns of targeted mutations in later generations were also examined. Mutations of most homozygous T_0_ plants were stably transmitted to the T_1_ generation (Table S4). However, unexpected genotypes were detected in the T_1_ generation of four of the five bi-allelic T_0_ plants. Additionally, a large proportion of the progeny of a chimeric T_0_ plant were chimeras (Tables S4 and S5). The transmission of mutations of several randomly selected T_1_ lines that did not contain transgenes (‘transgene-free’; see Section 2.2) in the T_2_ generation was also examined. The genotypes of all these lines were faithfully transmitted to T_2_ plants (Table S6).

### 2.2. Segregation of T-DNA in the T_1_ Generation

The presence of CRISPR/Cas9 DNA and marker genes in gene-edited plants may cause adverse effects, such as an increased risk of off-target changes, and may trigger regulation concerns when these plants are used in crop breeding [22,31,32]. To test whether the T-DNA fragment carrying the CRISPR/Cas9 construct could be segregated out in the progeny of T_0_ mutants, the presence of *HPT*, sgRNA, and *Cas9* transgenes in T_1_ plants derived from one of the homozygous or bi-allelic T_0_ mutants of each target (except Osfwl3a) was examined (Figure S2). For target Osfwl3a, the progeny of a chimeric T_0_ plant (Osfwl3a#4) were used. The genotype of the T_0_ mutant for each T_1_ line used is shown in Table S1. Transgene-free plants were obtained in several randomly selected T_1_ progeny for all lines (Figure S2), suggesting that the number of T-DNA insertion loci was low in T_0_ plants.

In most T_1_ lines (13 out of 15) analyzed, consistent segregation patterns were observed for all the *HPT*, sgRNA, and *Cas9* transgenes (Figure S2). However, inconsistent segregation patterns were detected in two lines (Osfwl3a#4 and Osfwl4b#6). In line Osfwl3a#4, *HPT* and *Cas9* sequences were not detected in some plants, although the sgRNA sequence was present (Figure S2). Interestingly, the eight T_1_ plants of this line that lacked the complete sgRNA/*Cas9* expression cassette were all homozygotes, whereas the other 12 plants containing the complete sgRNA/*Cas9* expression cassette were all chimeras (Figures S2 and S3; Tables S4 and S5). In line Osfwl4b#6, sgRNA and *Cas9* sequences were not detected in all examined T_1_ plants, but the *HPT* sequence was present in some of the plants (Figure S2). Next, we examined the T_0_ plant of this line for the presence of a T-DNA fragment and found that it also lacked sgRNA and *Cas9* sequences. This indicates that mutations of this line were generated by transient CRISPR/Cas9 expression.

### 2.3. Phenotypic Analysis of Rice FWL Gene Mutants

Phenotypes of allelic mutant lines that were homozygous, transgene-free, and with no detected off-target effects (see Section 2.4) of the *OsFWL1* and *OsFWL4* genes were analyzed. For *OsFWL1*, T_2_ plants of lines Osfwl1a#4 and Osfwl1b#11 (Table S6) were selected for Osfwl1a and Osfwl1b targets, respectively. For *OsFWL4*, T_3_ plants of lines Osfwl4a#7 and Osfwl4b#6 (Table S6) were analyzed for Osfwl4a and Osfwl4b targets, respectively.

**Figure 2:**
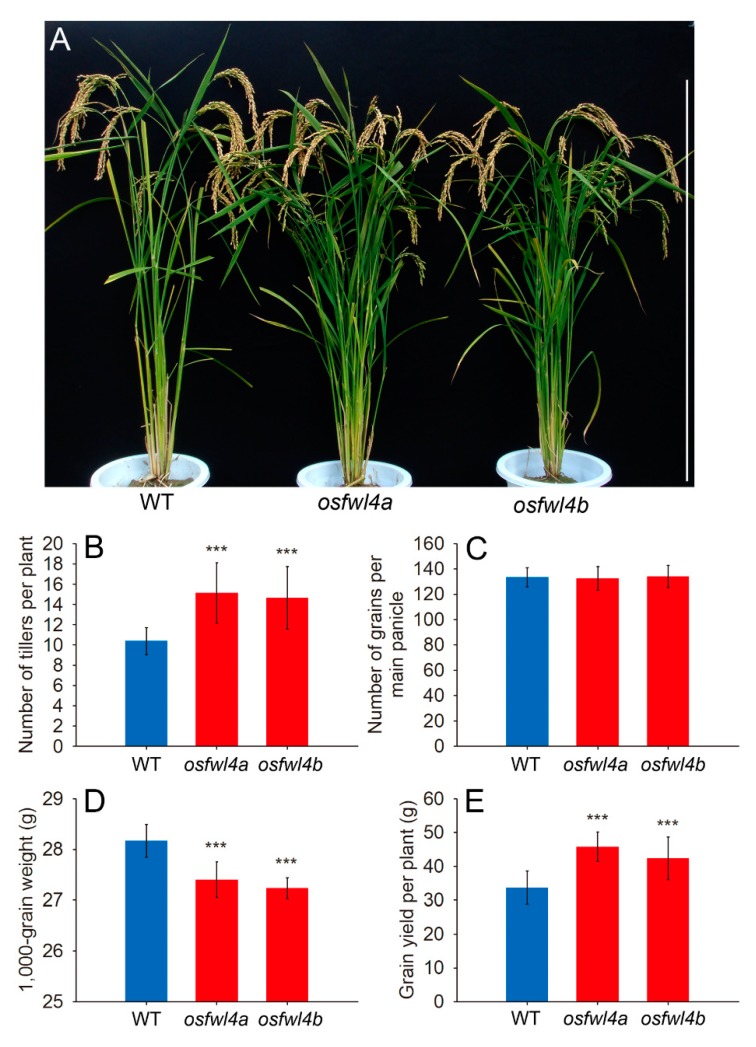
Analysis of yield traits of wild type (WT) and *OsFWL4* gene mutants. (**A**) WT and mutant plants, bar = 1 m. (**B**) Number of tillers per plant of the WT and mutants, *n* = 20. (**C**) Number of grains per main panicle of the WT and mutants, *n* = 10. (**D**) 1000-grain weight of the WT and mutants, *n* = 10. (**E**) Grain yield per plant of the WT and mutants, *n* = 12–15. Error bars are standard deviations. ***p < 0.001.

The number of tillers per *osfwl4a* and *osfwl4b* mutant plants was 45.9% and 41.1% greater, respectively, than that of the wild type (WT; Figure 2A,B). The number of grains per panicle of mutants was not significantly changed (Figure 2C). Although 1000-grain weight of mutants was slightly reduced (Figure 2D), the grain yield per plant was increased by 25.6–35.8% (Figure 2E).

**Figure 3:**
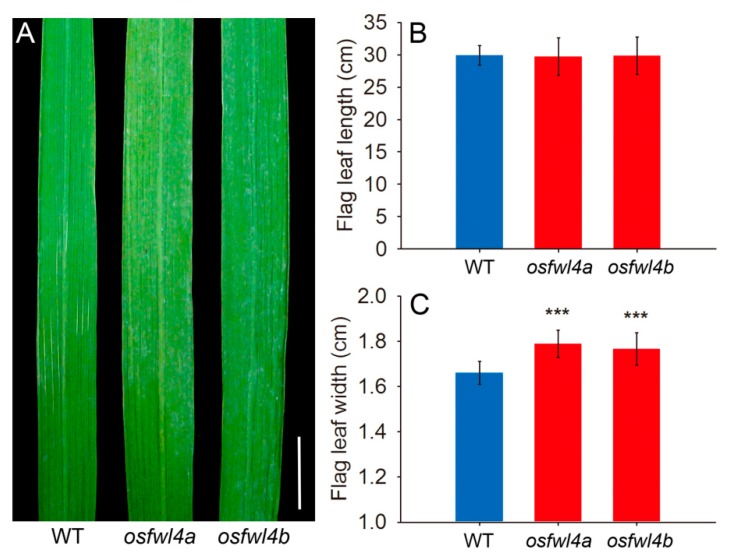
Phenotypes of flag leaves of WT and *OsFWL4* gene mutants. (**A**) Flag leaves of the WT and mutants, bar = 2 cm. (**B**) Flag leaf length of the WT and mutants. (**C**) Flag leaf width of the WT and mutants. The values in (**B**) and (**C**) are means of 20 plants. Error bars are standard deviations. ***p < 0.001.

Additionally, the flag leaf width of *osfwl4a* and *osfwl4b* mutants was 7.7% and 6.3% greater, respectively, than that of the WT (Figure 3A,C). However, there was no marked difference in flag leaf length (Figure 3B). Analysis of leaf epidermal cell size revealed no significant difference in cell length and width between WT and mutants (Figure S4). This suggests that the increase in leaf width of mutants was caused by an increase in cell number but not in cell size. In addition, the plant height of mutants was slightly reduced compared with that of the WT (Figure 2A and Figure S5).

**Figure 4:**
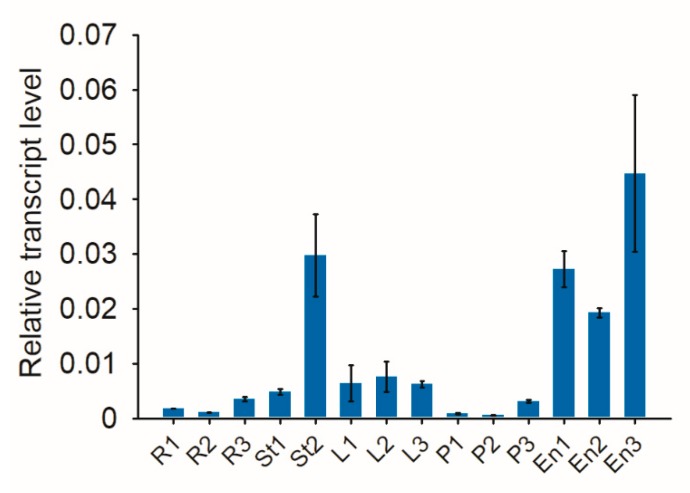
qRT-PCR results of the *OsFWL4* gene in 14 tissue samples of *japonica* rice Zhonghua 11. The rice *Actin1* gene was used as the internal control. Legend: R1–R3, roots in the seedling, tillering, and heading stages, respectively; St1 and St2, stems in the jointing and heading stages, respectively; L1–L3, leaves in the seedling, tillering, and heading stages, respectively; P1–P3, panicles 5, 15, and 20 cm in length, respectively; En1–En3, endosperms 5, 14, and 21 days after pollination. Error bars are standard deviations of three technical repeats.

The expression profile of the *OsFWL4* gene during the life cycle of rice was examined using qRT-PCR. *OsFWL4* was mainly expressed in the developing endosperm and the stem at the heading stage (Figure 4); it was also expressed in the leaf, root, and panicle. To gain insights into the molecular function of *OsFWL4*, a gene co-expression analysis was performed using the Genevestigator program [33] with the mRNA-Seq datasets. Many positively correlated genes of *OsFWL4* were involved in cell signal transduction, disease resistance, and heavy metal resistance (Table S7). Interestingly, some negatively correlated genes of *OsFWL4* were found to encode the F-box domain- and BTB (bric-a-brac, tramtrack and broad complex) domain-containing proteins, which may play a role in protein ubiquitination [34] (Table S8).

**Figure 5:**
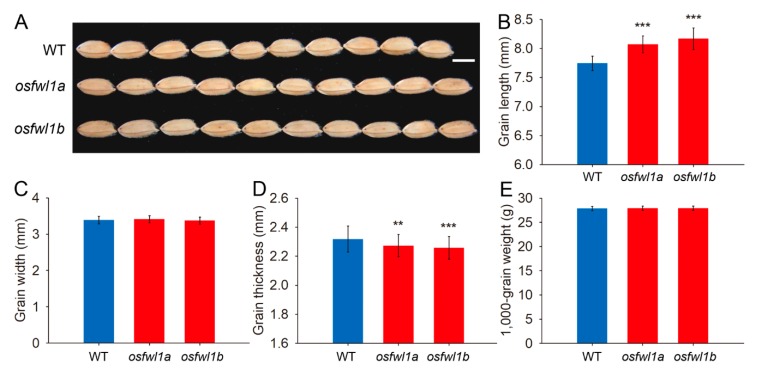
Analysis of grain shape of WT and *OsFWL1* gene mutants. (**A**) Grains of the WT and mutants, bar = 5 mm. (**B**) Grain length of the WT and mutants, *n* = 50. (**C**) Grain width of the WT and mutants, *n* = 50. (**D**) Grain thickness of the WT and mutants, *n* = 50. (**E**) 1000-grain weight of the WT and mutants, *n* = 10. Error bars are standard deviations. **p < 0.01; ***p < 0.001.

Grain length of *osfwl1a* and *osfwl1b* mutants was 4.2% and 5.5% greater, respectively, than that of the WT (Figure 5A,B). However, there was no difference in grain width between the mutants and WT plants (Figure 5C). Grain thickness of mutants was slightly lower than that of the WT (Figure 5D). Finally, there was no change in the 1000-grain weight of mutants (Figure 5E). Additionally, plant height, leaf size, and grain yield per plant of mutants were not considerably different from those of the WT (Figure S6).

### 2.4. Analysis of Off-Target Effects

To investigate the potential off-target events in our experiments, the two most probable off-target sites were selected for each of the 15 targets. The potential editing events in these sites were examined in all T_0_ plants and several randomly selected T_1_ lines. When the same off-target sequence occurred at different genomic loci, only one locus was examined. Of the 30 putative off-target sites, we detected mutations in 13 sites (Table 3). All the five loci that had a single-base mismatch with sgRNA exhibited off-target effects. Mutations were detected in four of the five (80.0%) and three of the 10 (30.0%) loci that had two and three mismatches, respectively. Additionally, one locus (Osfwl2aOFF-2) that had four dispersed mismatches was cleaved in transgenic plants. No mutations were detected in the loci that had five mismatches with sgRNA (Table 3). Most of the off-target sites with mutations were located within the gene region (Table 3).

Among the putative off-target sites with two mismatches, the mismatched nucleotides of Osfwl6bOFF-1 were separated by only one nucleotide in the PAM-proximal region, and no modifications were detected at this site (Table 3). Genotype analysis of the off-target mutations in T_0_ plants revealed that 44.9% and 43.5% of the genotypes were bi-alleles and heterozygotes, respectively (Figure 1C). Most off-target mutation events were insertions and deletions as observed in the on-target mutation events (Figure 1A,D).

### 2.5. Expression Analysis of Cas9 in Transgenic Plants

**Figure 6:**
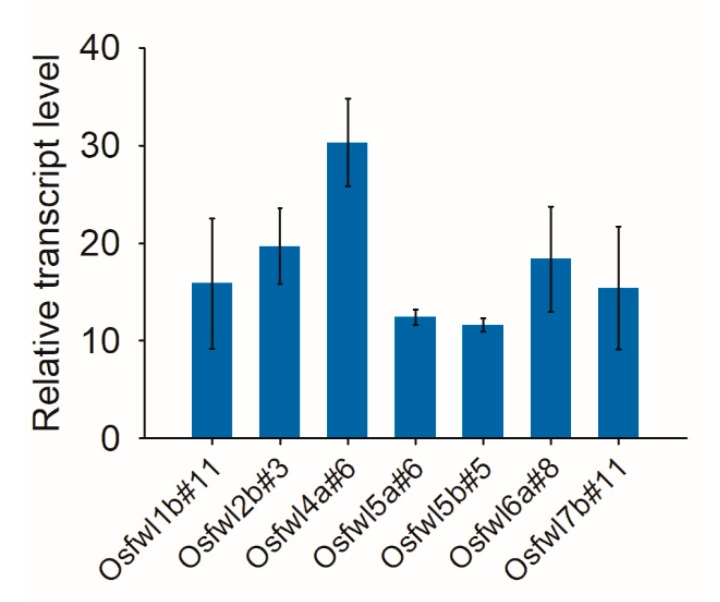
qRT-PCR analysis of *Cas9* expression in the transgene-positive plants of the T_1_ lines. The rice *Actin1* gene was used as the internal control. Error bars represent standard deviations of three biological repeats.

The expression level of *Cas9* was examined in transgene-positive plants of several randomly selected T_1_ lines by qRT-PCR. The *Cas9* mRNA level was approximately 11.6–30.3 fold that of the *OsActin1* gene in different lines (Figure 6).

## 3. Discussion

In this study, rice *FWL* family genes were mutated using the *Agrobacterium*-mediated CRISPR/Cas9 system, and the phenotypes of mutants of two genes (*OsFWL1* and *OsFWL4*) were characterized. The results suggest that the *OsFWL4* gene is a negative regulator of tiller number and plant yield in rice and that the *OsFWL1* gene plays a role in modulating rice grain length.

Rice tiller number is an important agronomic trait that largely affects grain yield. The tiller number of the *OsFWL4* gene mutants was increased by up to 45.9% compared with that of the WT (Figure 2A,B). Additionally, flag leaf width of mutants was also increased (Figure 3A,C). Leaf epidermal cell observation revealed that the increase in leaf width of mutants was caused by an increase in cell number but not in cell size (Figure S4). Hence, OsFWL4 may negatively affect cell proliferation during leaf and tiller development. In the mutants, the grain yield per plant was increased by up to 35.8% (Figure 2E), suggesting that the *OsFWL4* gene may be useful in breeding to improve rice yield.

The grain length of the *OsFWL1* gene mutants was significantly higher than that of the WT (Figure 5A,B). Similarly, grain length of the *OsFWL3* gene mutant has also been reported to be increased [9]. However, grain width of the *OsFWL1* gene mutants was not affected and grain thickness was reduced (Figure 5C,D). The decrease in grain thickness might be caused by insufficient grain filling due to enlarged glumes in the mutants. Finally, grain weight of mutants was not changed. Together, these results suggest that rice *FWL* genes play a role in the regulation of organ development in rice.

It has been reported that the *OsFWL4* gene can enhance Cd resistance when expressed in yeast cells and mediate the translocation of Cd from the roots to shoots in rice seedlings [15]. Recently, the *OsFWL1* gene was also reported to mediate Cd homeostasis in rice [20]. Hence, the two rice *FWL* genes function in both organ development regulation and Cd homeostasis in rice. Similarly, the *OsFWL5*/*PCR1* gene has been found to confer Cd resistance and Zn hypersensitivity upon expression in yeast and to modulate grain size and weight and metal ion homeostasis in rice [13,14]. However, how a single *FWL* gene fulfills such diverse roles remains unknown. Interestingly, the OsFWL5/PCR1 protein has been found to be localized as oligomers in the plasma membrane microdomains [13]. Additionally, GmFWL1, an important FWL protein involved in soybean nodulation, has also been demonstrated to be a plasma membrane microdomain-associated protein [5,10]. The plasma membrane microdomains are membrane sub-compartments consisting of special lipids and proteins and are considered signal integration hubs of cells [35]. Hence, the membrane microdomain-associated FWL protein may act in several distinct signaling pathways and thus affect multiple biological processes in plants. Both OsFWL1 and OsFWL4 proteins are located in the plasma membrane, and the OsFWL4 protein is distributed in a punctate manner [9,15]. We speculate that OsFWL4 may also be a microdomain-associated protein. A gene co-expression analysis revealed that *OsFWL4* may be involved in many cell functions (Tables S7 and S8). The *OsFWL1* gene is reported to be co-expressed with the zinc finger and ubiquitination-related protein genes [9].

CRISPR/Cas9 DNA can be delivered into rice cells by *Agrobacterium*-mediated transformation and integrated into the rice genome. Studies have shown that T-DNA truncation frequently occurs in *Agrobacterium*-mediated transformation [36,37]. Detection of CRISPR/Cas9 DNA in unmutated T_0_ plants revealed that most (25 out of 39, excluding two plants that escaped hygromycin selection) of them lacked sgRNA and/or *Cas9* transgenes (Table S2). This indicates that the integrity of the sgRNA/*Cas9* expression cassette is an important factor affecting editing efficiency. Truncation of T-DNA can occur at its different ends (left, right, or both ends) and different stages of integration (before or during integration) [36,37,38,39]. In rice, truncated T-DNAs were detected in more than 18% of the transformants [40]. Hence, improving the quality of T-DNA integration may aid in further increasing the efficiency of CRISPR/Cas9 gene editing based on *Agrobacterium*-mediated transformation. 

T-DNA segregation analysis revealed that transgene-free plants could be obtained in several T_1_ plants for all lines examined (Figure S2). This suggests that T-DNA insertions in CRISPR/Cas9 gene-edited plants can be easily eliminated in the T_1_ generation. Interestingly, inconsistent segregation of *HPT*, sgRNA, and *Cas9* transgenes was observed in two lines (Osfwl3a#4 and Osfwl4b#6; Figure S2). The absence of sgRNA and *Cas9* transgenes in Osfwl4b#6 T_1_ plants was caused by the lack of these sequences in the T_0_ plant. In the 20 T_1_ plants examined for line Osfwl3a#4, seven plants contained only the sgRNA transgene, 12 plants contained all the three transgenes, and one plant had no transgene (Figures S2 and S3). This inconsistent segregation could be attributed to the presence of two T-DNA insertion sites in this line; one contained the complete T-DNA fragment, whereas the other harbored a truncated T-DNA with only the sgRNA transgene.

The off-target effect is a major concern in the application of CRISPR/Cas9 technology. Several studies have reported that the CRISPR/Cas9 system is highly specific in plants [27,32,41,42]. However, moderate or even high-frequency off-target mutagenesis has also been reported [43,44,45,46]. In the present study, potential editing events at 30 putative off-targets of the 15 sgRNAs were examined. We detected mutations in 13 out of the 30 putative off-target sites (Table 3). Analysis of the relationship between mismatch numbers of target-like sequences and off-target activity revealed that all the sequences harboring single mismatches with the sgRNAs and 80.0% of the sequences containing double mismatches were cleaved. These results indicate that at least two mismatches between the sgRNA and potential off-target sequences are required to minimize the off-target effects. Interestingly, an off-target site with up to four mismatches (Osfwl2aOFF-2) was also mutated (Table 3). The first mismatch of this site located at the first base in the 5′ end is usually tolerated by CRISPR/Cas9. Additionally, all four mismatched bases of this site were adenine (Table 3), which led to rN:dT base pairing during sgRNA binding. Generally, the rN:dT mismatches are well tolerated [47,48]. Hence, both the identity and position of mismatched bases might contribute to the cleavage of this site by Cas9. The results suggest that the sgRNAs should be designed carefully to minimize or avoid off-target mutagenesis in plants.

## 4. Materials and Methods 

### 4.1. Plant Materials and Growth Conditions

The rice variety used for transformation was Zhonghua 11 (*Oryza sativa* L. ssp. *japonica*). The rice plants were grown in experimental fields of the Huaiyin Normal University in Huai’an, China or in Lingshui, China in different growing seasons. Rice plants were also grown in plastic buckets in growth chambers with a 14/10 h light/dark cycle at 30 and 25 °C.

### 4.2. Construction of the CRISPR/Cas9 Plasmids

Maize ubiquitin promoter was used to drive the expression of the *hSpCas9* gene, which was amplified from the pX260 vector [49]. The construct was inserted into the pCAMBIA1300 vector (Cambia, Canberra, Australia) harboring the hygromycin resistance gene. The *Bsa*I site originally present in the pCAMBIA1300 vector was disrupted by point mutation. Subsequently, a construct containing the OsU6 promoter [50], a negative selection marker gene (*ccdB*) with *Bsa*I sites at both ends, and a fragment encoding the sgRNA scaffold derived from the pX260 vector was cloned into this vector to generate the CRISPR/Cas9 binary vector (Figure S1). Target sequences containing at least one mismatch in the 12-bp PAM proximal region with other genomic sites and relatively high GC content were selected for the rice *FWL* genes. The designed target sequences were synthesized, annealed, and ligated into the *Bsa*I site of the CRISPR/Cas9 binary vector to obtain the CRISPR plasmids for targeted gene editing. The plasmids were propagated in *Escherichia coli* competent cells and subsequently introduced into the *Agrobacterium tumefaciens* strain EHA105 for *Agrobacterium*-mediated transformation of rice [51].

### 4.3. Detection of On-Target and Off-Target Mutations

The potential off-targets of sgRNAs were predicted using the “offTarget” program in the CRISPR-GE software toolkit [52]. Genomic DNA of rice was extracted using the CTAB (cetyl trimethylammonium bromide) method. The DNA fragments covering the on-target and off-target sites were amplified by PCR using the specific genomic primers. PCR amplifications were performed in a Mastercycler nexus gradient thermal cycler (Eppendorf, Hamburg, Germany). Each reaction contained DNA templates, 1 × PCR buffer, 0.4 mmol L^−1^ dNTPs (deoxynucleotide triphosphates), 0.3 μmol L^−1^ of both forward and reverse primers, and 1 U KOD FX DNA polymerase (Toyobo, Osaka, Japan). Distilled water was added to a final volume of 50 μL. The PCR conditions included an initial incubation at 94 °C for 2 min, followed by 30 cycles of 98 °C for 10 s, 50–55 °C for 30 s, and 68 °C for 0.5–1 min, with a final extension at 68 °C for 5 min. The amplified products were sequenced directly. For some samples, PCR products were cloned and individual clones were sequenced. The superimposed sequencing chromatograms of heterozygous and bi-allelic mutations were decoded using DSDecodeM [53]. The PCR primers used are listed in Table S9.

### 4.4. Detection of the T-DNA Fragment

The T-DNA fragment in transgenic plants was detected by PCR using three pairs of primers amplifying the *HPT*, sgRNA, and *Cas9* transgenes. Amplifications were carried out in a Mastercycler nexus gradient thermal cycler (Eppendorf, Hamburg, Germany). Each reaction contained DNA templates, 1 × Es Taq MasterMix (Cwbio, Beijing, China), and 0.4 μmol L^−1^ of both forward and reverse primers. Distilled water was added to a final volume of 25 μL. The PCR conditions included an initial incubation at 94 °C for 2 min, followed by 30 cycles of 94 °C for 30 s, 55 °C for 30 s, and 72 °C for 30 s, with a final extension at 72 °C for 2 min. PCR products were separated by electrophoresis on 1.5% agarose gels containing GoldView I nucleic acid dye (Solarbio, Beijing, China). The primers used are listed in Table S9.

### 4.5. RNA Isolation and qRT-PCR

RNA isolation and qRT-PCR analysis were performed as previously described [54]. Briefly, total RNA was isolated using the TRIzol Total RNA Isolation kit (Sangon Biotech, Shanghai, China) and treated with DNase I (Sangon Biotech, Shanghai, China). Eight hundred nanograms of total RNA was reverse-transcribed using RevertAid Premium Reverse Transcriptase (Thermo Fisher Scientific, Waltham, MA, USA) and diluted ten-fold for PCR amplification. The PCR was performed on a LightCycler480 II instrument (Roche, Basel, Switzerland). Each reaction contained 2 μL of cDNA template, 10 μL of SYBR Green qPCR Master Mix (BBI, Toronto, ON, Canada), and 0.2 μmol L^−1^ gene-specific primers in a final volume of 20 μL. The PCR conditions included an initial incubation at 95 °C for 3 min, followed by 45 cycles of 95 °C for 5 s and 60 °C for 30 s. The specificity of the PCR reactions was determined by melting curve analyses of the products. Relative expression levels were calculated by the 2^−ΔΔCT^ method. The rice *Actin1* gene was used as the internal control. The primer sequences are listed in Table S9.

### 4.6. Leaf Epidermal Cell Observation

Epidermal cells in flag leaves were observed following the method used by Yoshikawa et al. [55]. 

### 4.7. Trait Measurement

Plant height, leaf size, and tiller number of WT and mutants were measured in the field at the maturity stage. For tiller number determination, only seed setting tillers were counted. Rice plants were harvested when the grains were fully mature. Grains threshed from each plant were dried, and filled grains were weighed to determine grain yield per plant. Fully filled grains were used for determining grain size and weight. Grain weight was measured based on 100 grains and converted to 1000-grain weight.

## 5. Conclusions

Collectively, our findings showed that transgene-free rice plants with targeted mutations can be produced in the T_1_ generation using the *Agrobacterium*-mediated CRISPR/Cas9 system, and that the *OsFWL4* gene plays a role in the regulation of tillering and plant yield in rice. The specific mutants obtained in this study provide valuable materials for functional analysis of rice *FWL* genes.

## Figures and Tables

**Table 1:** Target sites of rice *FWL* genes for clustered regularly interspaced short palindromic repeats (CRISPR)/CRISPR-associated protein 9 (Cas9)-mediated gene editing.

Gene	Locus	Target Name	Target Sequence (5′–3′) ^1^	GC Content (%)
*OsFWL1*	LOC_Os02g52550	Osfwl1a	CTGAAGGACTTACAGTTTCC GGG	45
		Osfwl1b	TGGGCAGGTCGCTGACATCG TGG	65
*OsFWL2*	LOC_Os02g36940	Osfwl2a	GCGCTGGTGATGCTCCTCAC GGG	65
		Osfwl2b	CATCTTGGCGCGGTAGAAGC AGG	60
*OsFWL3*	LOC_Os02g36950	Osfwl3a	ATCGCGGAGATCGTCGACCG GGG	65
		Osfwl3b	GTGGACGAGGCAGTCGGGGC AGG	75
*OsFWL4*	LOC_Os03g61440	Osfwl4a	ATTGAAGCAGGCGAAGAGTC CGG	50
		Osfwl4b	CGCAGCATGGGTCCTCGGGG AGG	75
*OsFWL5*	LOC_Os10g02300	Osfwl5a	ATCGCAGAAATCGTCGACAG GGG	50
		Osfwl5b	CTCACGGTGCATCTGTGCGA TGG	60
*OsFWL6*	LOC_Os03g61470	Osfwl6a	TCGACGTCGTGCGGCACCGG CGG	75
		Osfwl6b	GGCAAGATGCGCACTCAGTA CGG	55
*OsFWL7*	LOC_Os03g61500	Osfwl7a	CCCGTGCATCACGTTCGGGC GGG	70
		Osfwl7b	CATCTTGCCCCGGTAGACGC AGG	65
*OsFWL8*	LOC_Os03g61480	Osfwl8a	GGGTCGACGTCGTTCGGCAC CGG	70
		Osfwl8b	GTTGAGGTGCCATCCGAGCT TGG	60

^1^ The protospacer adjacent motif (PAM) sequences are shown in green.

**Table 2:** Identification of targeted mutations in T_0_ plants.

Target	No. of T_0_ Plants Obtained	No. of Plants with Mutations	Zygosity				Combined Percentage of Homozygous and Bi-Allelic Mutants (%)
			Homozygous	Bi-Allelic	Heterozygous	Chimeric	
Osfwl1a	15	7 (46.7%)	–	7 (46.7%)	–	–	46.7
Osfwl1b	14	14 (100.0%)	9 (64.3%)	5 (35.7%)	–	–	100.0
Osfwl2a	16	15 (93.8%)	1 (6.3%)	14 (87.5%)	–	–	93.8
Osfwl2b	14	11 (78.6%)	3 (21.4%)	8 (57.1%)	–	–	78.6
Osfwl3a	7	6 (85.7%)	–	4 (57.1%)	–	2 (28.6%)	57.1
Osfwl3b	25	22 (88.0%)	2 (8.0%)	13 (52.0%)	1 (4.0%)	6 (24.0%)	60.0
Osfwl4a	15	11 (73.3%)	3 (20.0%)	8 (53.3%)	–	–	73.3
Osfwl4b	15	10 (66.7%)	1 (6.7%)	8 (53.3%)	–	1 (6.7%)	60.0
Osfwl5a	14	14 (100.0%)	2 (14.3%)	12 (85.7%)	–	–	100.0
Osfwl5b	14	14 (100.0%)	7 (50.0%)	7 (50.0%)	–	–	100.0
Osfwl6a	15	4 (26.7%)	1 (6.7%)	3 (20.0%)	–	–	26.7
Osfwl6b	16	15 (93.8%)	5 (31.3%)	10 (62.5%)	–	–	93.8
Osfwl7a	14	13 (92.9%)	2 (14.3%)	10 (71.4%)	1 (7.1%)	–	85.7
Osfwl7b	15	12 (80.0%)	5 (33.3%)	6 (40.0%)	1 (6.7%)	–	73.3
Osfwl8b	14	14 (100.0%)	3 (21.4%)	11 (78.6%)	–	–	100.0
Total	223	182 (81.6%)	44 (19.7%)	126 (56.5%)	3 (1.3%)	9 (4.0%)	76.2

**Table 3:** Off-target effect analysis of transgenic plants.

Name of Putative Off-Target Site	Locus	Sequence of Putative Off-Target Site ^1^	Region	No. of Mismatching Bases	No. of Plants Tested	No. of Plants with Mutations
Osfwl1aOFF-1	Chr2: 11000661–11000683	CTGAATGACTGACTGTCTCC TGG	LOC_Os02g18850 intron	4	32	0
Osfwl1aOFF-2	Chr2: 1853752–1853774	CTGAAGGACTTGCACATTTC AGG	LOC_Os02g04230 intron	4	32	0
Osfwl1bOFF-1	Chr6: 17267236–17267258	CGGGCAGGACGCCGACATCG CGG	LOC_Os06g29994 CDS	3	32	2
Osfwl1bOFF-2	Chr8: 15832268–15832290	TGTGCAGGTCGATGACATCA TGG	Intergenic	3	32	0
Osfwl2aOFF-1	Chr1: 14111612–14111634	GCGATGGTGATGCTCCTCGC CGG	Intergenic	2	32	4
Osfwl2aOFF-2	Chr6: 463763–463785	ACGCAGGTGAAGCTCCTAAC TGG	LOC_Os06g01800 intron	4	32	7
Osfwl2bOFF-1	Chr4: 24327403–24327425	CATCTTGGGGAGGTAGAAGA AGG	LOC_Os04g40990 CDS	3	30	0
Osfwl2bOFF-2	Chr2: 22320818–22320840	CAGCTTGGAGCGGTAGATGC AGG	LOC_Os02g36950 CDS	3	30	0
Osfwl3aOFF-1	Chr4: 23060613–23060635	ATCGCGGAGATCGTCGACCA GGG	LOC_Os04g38790 CDS	1	27	26
Osfwl3aOFF-2	Chr2: 22312627–22312649	ATCGCGGAGATCATCGACCG GGG	LOC_Os02g36940 CDS	1	27	26
Osfwl3bOFF-1	Chr2: 22312479–22312501	GTGGACGGGGCAGTCGGCGC AGG	LOC_Os02g36940 CDS	2	41	7
Osfwl3bOFF-2	Chr2: 4426323–4426345	GTGGAAGAAGAAGTCGAGGC AGG	LOC_Os02g08330 CDS	4	41	0
Osfwl4aOFF-1	Chr7: 3799011–3799033	TTTGAAGCAGGTGAAGAGTC CGG	LOC_Os07g07580 intron	2	28	23
Osfwl4aOFF-2	Chr9: 2298486–2298508	CATGAGGAAGGCGAGGAGTC CGG	LOC_Os09g04339 CDS	5	28	0
Osfwl4bOFF-1	Chr1: 31161078–31161100	CGCTGCATCTGTCCTCGGGA AGG	Intergenic	4	31	0
Osfwl4bOFF-2	Chr2: 5315090–5315112	AGCAGAAAGGATCCTGGGGG AGG	Intergenic	5	31	0
Osfwl5aOFF-1	Chr6: 7393721–7393743	ATCTCAGAAATAATCGACAG CGG	Intergenic	3	31	0
Osfwl5aOFF-2	Chr4: 19176717–19176739	GTCCCAGGACTCGTCGACAG AGG	LOC_Os04g32020 5′ UTR	4	31	0
Osfwl5bOFF-1	Chr4: 34131133–34131155	CGCCCGGTGCATCTGCGCGA TGG	LOC_Os04g57330 5′ UTR	3	32	3
Osfwl5bOFF-2	Chr6: 22532424–22532446	ATCACGGTGAGCATGTGCGA TGG	LOC_Os06g38090 intron	5	32	0
Osfwl6aOFF-1	Chr3: 34884082–34884104	TCGACGTCGTGCGGCACCAG CGG	LOC_Os03g61500 CDS	1	33	21
Osfwl6aOFF-2	Chr3: 16449514–16449536	AAGACGTCGAGCGGCACCGG CGG	LOC_Os03g28980 CDS	3	33	21
Osfwl6bOFF-1	Chr3: 34878541–34878563	GGCAAGATGCGCGCACAGTA CGG	LOC_Os03g61490 CDS	2	31	0
Osfwl6bOFF-2	Chr1: 29494129–29494151	AGCTAGACGTGCAATCAGTA CGG	Intergenic	5	31	0
Osfwl7aOFF-1	Chr3: 34870990–34871012	CCCGTGCATCACGTTCGGGA GGG	LOC_Os03g61470 CDS	1	31	17
Osfwl7aOFF-2	Chr10: 21731587–21731609	CCCATGCATCACGTTAGGTC CGG	LOC_Os10g40580 5′ UTR	3	31	0
Osfwl7bOFF-1	Chr11: 6506399–6506421	GATCTTGCTCCGGTCGACGC CGG	Intergenic	3	32	0
Osfwl7bOFF-2	Chr2: 22312530–22312552	CATCTTGGCGCGGTAGAAGC AGG	LOC_Os02g36940 CDS	3	32	0
Osfwl8bOFF-1	Chr3: 34883868–34883890	GTTGAGGTCCCATCCGAGCT TGG	LOC_Os03g61500 CDS	1	30	30
Osfwl8bOFF-2	Chr3: 34857174–34857196	GTTGAGGTGCCACCCAAGCT TGG	LOC_Os03g61430 CDS	2	30	13

^1^ The PAM sequences are shown in green and the mismatched bases in red.

## Supplementary Materials

Supplementary materials can be found at https://www.mdpi.com/1422-0067/21/3/809/s1.
