# Supplementary material for: Targeted Mutagenesis of the Rice FW 2.2-Like Gene Family Using the CRISPR/Cas9 System Reveals OsFWL4 as a Regulator of Tiller Number and Plant Yield in Rice
Source: Int J Mol Sci. 2020 Jan 26;21(3):809. doi: 10.3390/ijms21030809 (PMC7037146; doi:10.3390/ijms21030809)
Supplement: Supplementary file 1 [file ijms-21-00809-s001.pdf]

Supplementary Materials

Targeted Mutagenesis of the Rice *FW 2.2*-Like Gene Family Using the CRISPR/Cas9 System Reveals OsFWL4 as a Regulator of Tiller Number and Plant Yield in Rice

Qingsong Gao [1,†], Gang Li [2,†], Hui Sun [1], Ming Xu [1], Huanhuan Wang [1], Jianhui Ji [1], Di Wang [2], Caiyong Yuan [2,*] and Xiangxiang Zhao [1,*]

1 Jiangsu Collaborative Innovation Center of Regional Modern Agriculture & Environmental Protection/ Jiangsu Key Laboratory for Eco-Agricultural Biotechnology around Hongze Lake, Huaiyin Normal University, Huai'an 223300, China

2 Huaiyin Institute of Agricultural Sciences of Xuhuai Region in Jiangsu, Huai'an 223001, China

* Correspondence: xxzhao2013@163.com (X.Z.); hysdycy@163.com (C.Y.); Tel.: +86-517-83525885 (X.Z.); +86-517-83659907 (C.Y.)

† These authors contributed equally to this work

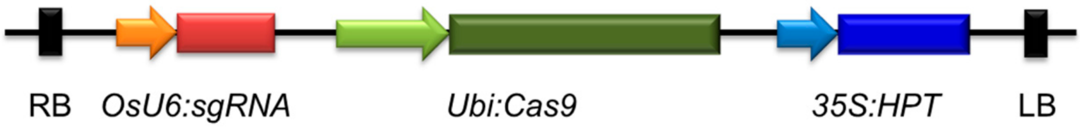

Figure S1. Schematic diagram of T-DNA region of the CRISPR/Cas9 vector. RB, right border; LB, left border.

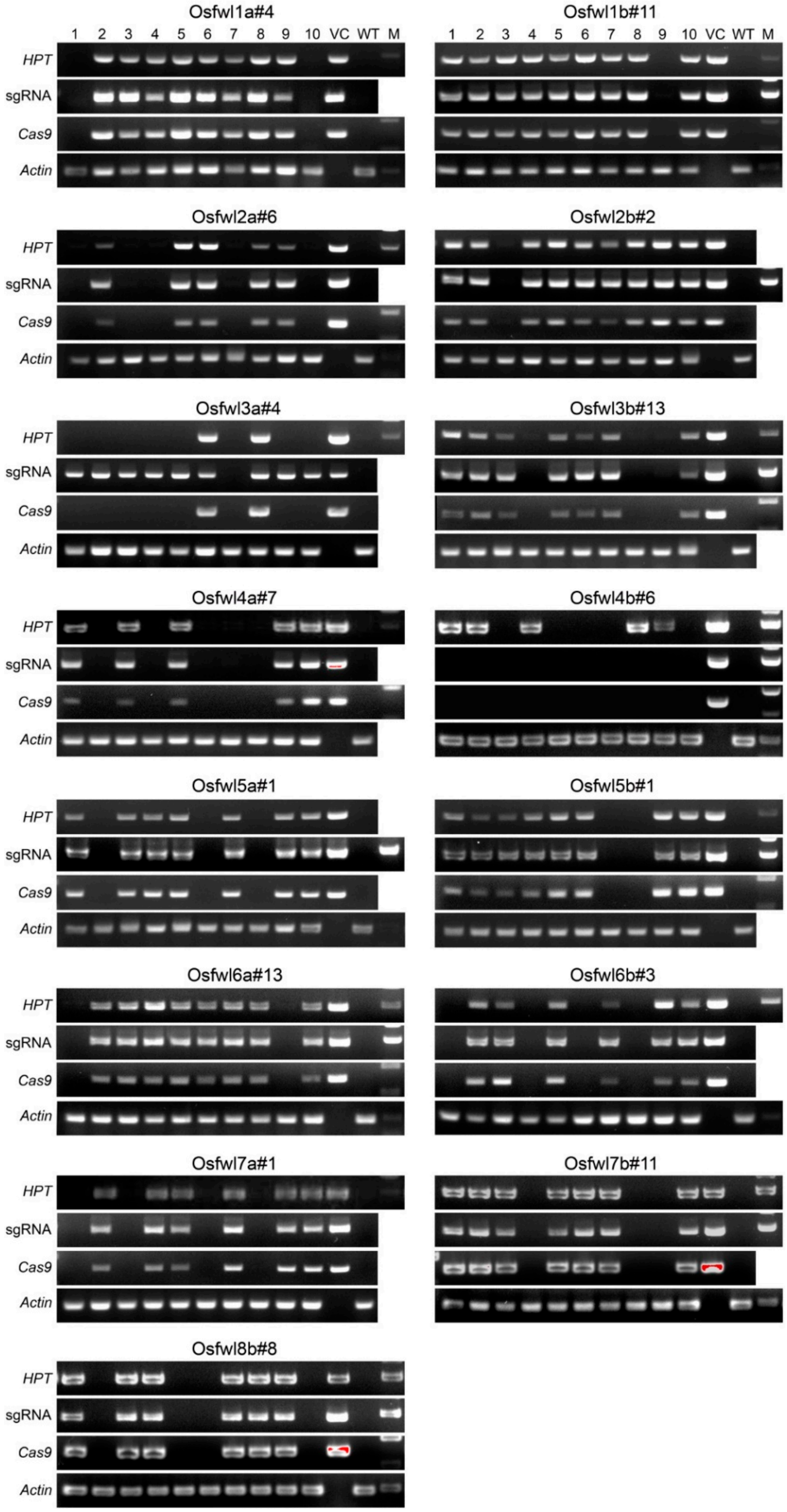

Figure S2. Segregation analysis of transgenes in T_1 mutant lines. One T_1 mutant line was selected for each target. The numbers 1–10 indicate different plants. VC, vector control; WT, wild type; M, DL2000 marker.

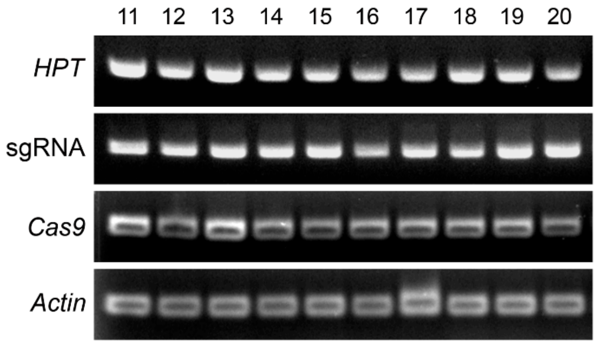

Figure S3. Detection of transgenes in additional 10 T_1 plants of line Osfwl3a#4. All these plants contained the three transgenes.

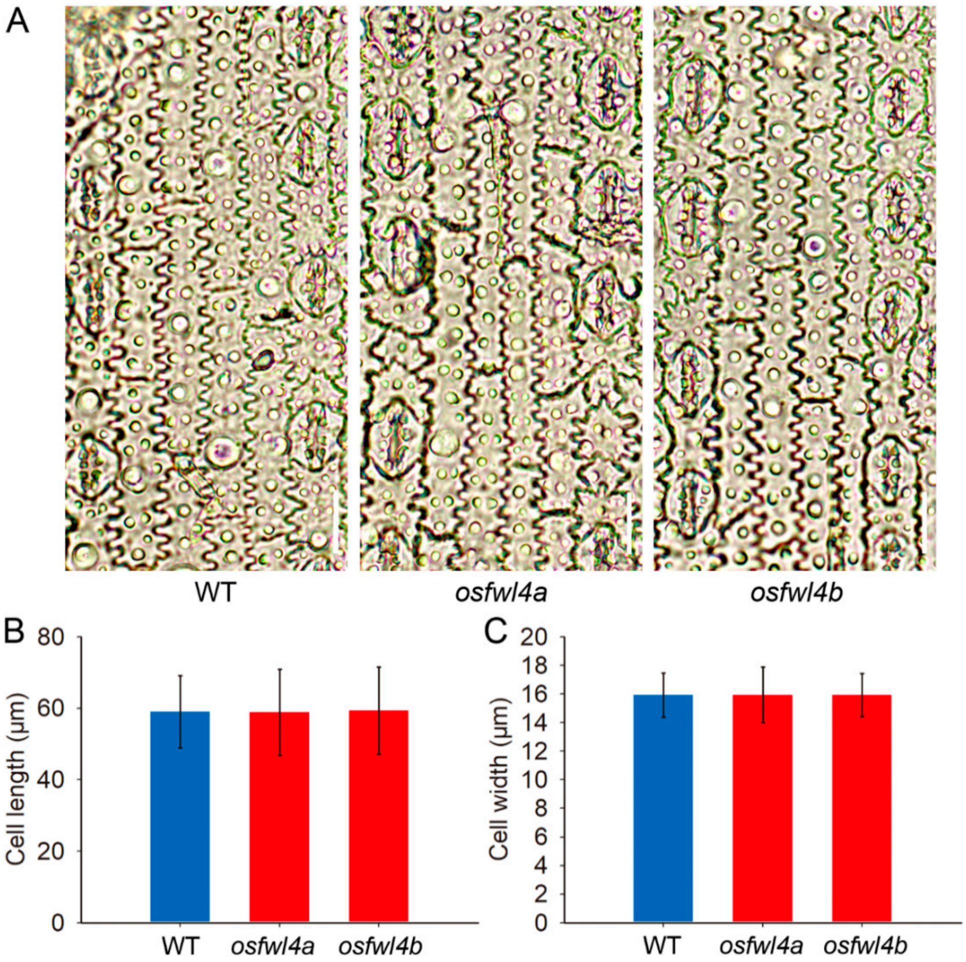

Figure S4. Epidermal cell size analysis of flag leaves of the wild type (WT) and *OsFWL4* gene mutants. (**A**) Leaf epidermal cells of the WT and *OsFWL4* gene mutants, bar = 20 μm. (**B**) Measurement of epidermal cell length of flag leaves in the WT and mutants. (**C**) Measurement of epidermal cell width of flag leaves in the WT and mutants. Error bars in (**B**) and (**C**) are standard deviations of 100 cells.

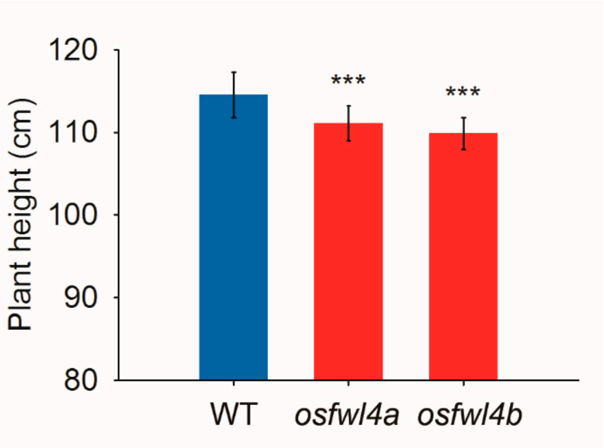

Figure S5. Plant height of the wild type (WT) and *OsFWL4* gene mutants. Error bars are standard deviations of 15 plants. *** $P < 0.001$.

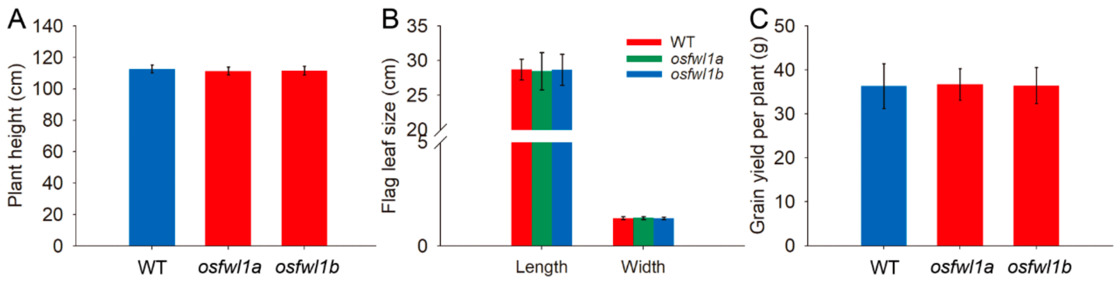

Figure S6. Phenotypes of wild type (WT) and *OsFWL1* gene mutants. (**A**) Plant height of WT and *OsFWL1* gene mutants, n = 10. (**B**) Flag leaf length and width of the WT and *OsFWL1* gene mutants. The values are means of 10–12 plants. (**C**) Grain yield per plant of WT and *OsFWL1* gene mutants, n = 10. Error bars are standard deviations.

Table S1. Targeted genomic mutations induced by the clustered regularly interspaced short palindromic repeats (CRISPR)/CRISPR-associated protein 9 (Cas9) system in the T_0 plants. The target sites are marked in red and the protospacer adjacent motif (PAM) sequences are underlined. The inserted or substituted nucleotides are shown in green. The plant name and zygosity are in bold font. Ref, reference sequence; Al#, different alleles; WT, wild type; i#, number of bp inserted; i#a/b, same number of different nucleotides inserted in the same site; d#, number of bp deleted; d#a/b, deletion of the same number of nucleotides at different sites; s#, number of bp substituted.

Osfwl1a#3, bi-allele

		Genotype
Ref	TTTCCACTGCATGGACGATCCCGGA-AACTGTAAGTCCTTCAGTCAGTCAGTTTTCCCACA	WT
Al1	TTTCCACTGCATGGACGATCCCGGAAAACTGTAAGTCCTTCAGTCAGTCAGTTTTCCCACA	i1a
Al2	TTTCCACTGCATGGACGATCCCGGATAACTGTAAGTCCTTCAGTCAGTCAGTTTTCCCACA	i1b

Osfwl1a#4, bi-allele

		Genotype
Ref	TTTCCACTGCATGGACGATCCCGGA-AACTGTAAGTCCTTCAGTCAGTCAGTTTTCCCACA	WT
Al1	TTTCCACTGCATGGACGATCCCGGATAACTGTAAGTCCTTCAGTCAGTCAGTTTTCCCACA	i1
Al2	TTTCCACTGCATGGACGATCCCGGA--ACTGTAAGTCCTTCAGTCAGTCAGTTTTCCCACA	d1

Osfwl1a#10, bi-allele

		Genotype
Ref	TTTCCACTGCATGGACGATCCCGGA-AACTGTAAGTCCTTCAGTCAGTCAGTTTTCCCACA	WT
Al1	TTTCCACTGCATGGACGATCCCGGATAACTGTAAGTCCTTCAGTCAGTCAGTTTTCCCACA	i1
Al2	TTTCCACTGCATGGACGATCCCGGA--ACTGTAAGTCCTTCAGTCAGTCAGTTTTCCCACA	d1

Osfwl1a#11, bi-allele

		Genotype
Ref	TTTCCACTGCATGGACGATCCCGGA-AACTGTAAGTCCTTCAGTCAGTCAGTTTTCCCACA	WT
Al1	TTTCCACTGCATGGACGATCCCGGATAACTGTAAGTCCTTCAGTCAGTCAGTTTTCCCACA	i1
Al2	TTTCCACTGCATGGACGATCCCGGA--ACTGTAAGTCCTTCAGTCAGTCAGTTTTCCCACA	d1

Osfwl1a#12, bi-allele

		Genotype
Ref	TTTCCACTGCATGGACGATCCCGGA-AACTGTAAGTCCTTCAGTCAGTCAGTTTTCCCACA	WT
Al1	TTTCCACTGCATGGACGATCCCGGAAAACTGTAAGTCCTTCAGTCAGTCAGTTTTCCCACA	i1
Al2	TTTCCACTGCATGGACGATCCCGGA--ACTGTAAGTCCTTCAGTCAGTCAGTTTTCCCACA	d1

Osfwl1a#13, bi-allele

		Genotype
Ref	TTTCCACTGCATGGACGATCCCGGA-AACTGTAAGTCCTTCAGTCAGTCAGTTTTCCCACA	WT
Al1	TTTCCACTGCATGGACGATCCCGGATAACTGTAAGTCCTTCAGTCAGTCAGTTTTCCCACA	i1
Al2	TTTCCACTGCATGGACGATCCCGGA--ACTGTAAGTCCTTCAGTCAGTCAGTTTTCCCACA	d1

Osfwl1a#14, bi-allele

Genotype

```
Ref     TTTCCACTGCATGGACGATCCCGGA-AACTGTAAGTCCTTCAGTCAGTCAGTTTTCCCACA    WT
Al1     TTTCCACTGCATGGACGATCCCGGAAAACTGTAAGTCCTTCAGTCAGTCAGTTTTCCCACA    i1
Al2     TTTCCACTGCATGGACGATCCCGGA---CTGTAAGTCCTTCAGTCAGTCAGTTTTCCCACA    d2
```

Osfwl1b#1, bi-allele

```
                                                                  Genotype
Ref     TGTGCCCCTGCATCACCTTTGGGCAGGTCGCTGACATCGTGGACAAGGGCACCTGCCGTG    WT
Al1     TGTGCCCCTGCATCACCTTTGGGCAGGTCGCTGACA-CGTGGACAAGGGCACCTGCCGTG    d1
Al2     TGTGCCCCTGCATCACC-------------------TCGTGGACAAGGGCACCTGCCGTG    d19
```

Osfwl1b#2, homozygote

```
                                                                  Genotype
Ref     TGTGCCCCTGCATCACCTTTGGGCAGGTCGCTGACATCGTGGACAAGGGCACCTGCCGTG    WT
Al1     TGTGCCCCTGCATCACCTTTGGGCAGGTCGCTGAC-TCGTGGACAAGGGCACCTGCCGTG    d1
Al2     TGTGCCCCTGCATCACCTTTGGGCAGGTCGCTGAC-TCGTGGACAAGGGCACCTGCCGTG    d1
```

Osfwl1b#3, homozygote

```
                                                                  Genotype
Ref     TGTGCCCCTGCATCACCTTTGGGCAGGTCGCTGACATCGTGGACAAGGGCACCTGCCGTG    WT
Al1     TGTGCCCCTGCATCACCTTTGGGCAGGTCGCTGAC-TCGTGGACAAGGGCACCTGCCGTG    d1
Al2     TGTGCCCCTGCATCACCTTTGGGCAGGTCGCTGAC-TCGTGGACAAGGGCACCTGCCGTG    d1
```

Osfwl1b#4, bi-allele

```
                                                                  Genotype
Ref     TGTGCCCCTGCATCACCTTTGGGCAGGTCGCTGACATCGTGGACAAGGGCACCTGCCGTG    WT
Al1     TGTGCCCCTGCATCACCTTTGGGCAGGTCGCTGACA-CGTGGACAAGGGCACCTGCCGTG    d1
Al2     TGTGCCCCTGCATCACC-------------------TCGTGGACAAGGGCACCTGCCGTG    d19
```

Osfwl1b#5, bi-allele

```
                                                                  Genotype
Ref     TGTGCCCCTGCATCACCTTTGGGCAGGTCGCTGACA-TCGTGGACAAGGGCACCTGCCGTG    WT
Al1     TGTGCCCCTGCATCACCTTTGGGCAGGTCGCTGACAATCGTGGACAAGGGCACCTGCCGTG    i1
Al2     TGTGCCCCTGCATCACCTTTGGGCAGG----------TCGTGGACAAGGGCACCTGCCGTG    d9
```

Osfwl1b#6, bi-allele

```
                                                                  Genotype
Ref     TGTGCCCCTGCATCACCTTTGGGCAGGTCGCTGACATCGTGGACAAGGGCACCTGCCGTG    WT
Al1     TGTGCCCCTGCATCACCTTTGGGCAGGTCGCTGAC-TCGTGGACAAGGGCACCTGCCGTG    d1
```

```
Al2    TGTGCCCCTGCATCACCTTTGGGCAGGTCGCTGA--TCGTGGACAAGGGCACCTGCCGTG    d2
```

Osfwl1b#7, homozygote

```
                                                                    Genotype
Ref    TGTGCCCCTGCATCACCTTTGGGCAGGTCGCTGACATCGTGGACAAGGGCACCTGCCGTG    WT
Al1    TGTGCCCCTGCATCACCTTTGGGCAGGTCGCTGAC-TCGTGGACAAGGGCACCTGCCGTG    d1
Al2    TGTGCCCCTGCATCACCTTTGGGCAGGTCGCTGAC-TCGTGGACAAGGGCACCTGCCGTG    d1
```

Osfwl1b#8, homozygote

```
                                                                    Genotype
Ref    TGTGCCCCTGCATCACCTTTGGGCAGGTCGCTGACATCGTGGACAAGGGCACCTGCCGTG    WT
Al1    TGTGCCCCTGCATCACCTTTGGGCAGGTCGCTGAC-TCGTGGACAAGGGCACCTGCCGTG    d1
Al2    TGTGCCCCTGCATCACCTTTGGGCAGGTCGCTGAC-TCGTGGACAAGGGCACCTGCCGTG    d1
```

Osfwl1b#9, homozygote

```
                                                                    Genotype
Ref    TGTGCCCCTGCATCACCTTTGGGCAGGTCGCTGACATCGTGGACAAGGGCACCTGCCGTG    WT
Al1    TGTGCCCCTGCATCACCTTTGGGCAGGTCGCTGAC-TCGTGGACAAGGGCACCTGCCGTG    d1
Al2    TGTGCCCCTGCATCACCTTTGGGCAGGTCGCTGAC-TCGTGGACAAGGGCACCTGCCGTG    d1
```

Osfwl1b#10, homozygote

```
                                                                    Genotype
Ref    TGTGCCCCTGCATCACCTTTGGGCAGGTCGCTGACATCGTGGACAAGGGCACCTGCCGTG    WT
Al1    TGTGCCCCTGCATCACCTTTGGGCAGGTCGCTGAC-TCGTGGACAAGGGCACCTGCCGTG    d1
Al2    TGTGCCCCTGCATCACCTTTGGGCAGGTCGCTGAC-TCGTGGACAAGGGCACCTGCCGTG    d1
```

Osfwl1b#11, homozygote

```
                                                                    Genotype
Ref    TGTGCCCCTGCATCACCTTTGGGCAGGTCGCTGACATCGTGGACAAGGGCACCTGCCGTG    WT
Al1    TGTGCCCCTGCATCACCTTTGGGCAGGTCGCTGAC-TCGTGGACAAGGGCACCTGCCGTG    d1
Al2    TGTGCCCCTGCATCACCTTTGGGCAGGTCGCTGAC-TCGTGGACAAGGGCACCTGCCGTG    d1
```

Osfwl1b#12, homozygote

```
                                                                    Genotype
Ref    TGTGCCCCTGCATCACCTTTGGGCAGGTCGCTGACATCGTGGACAAGGGCACCTGCCGTG    WT
Al1    TGTGCCCCTGCATCACCTTTGGGCAGGTCGCTGAC-TCGTGGACAAGGGCACCTGCCGTG    d1
Al2    TGTGCCCCTGCATCACCTTTGGGCAGGTCGCTGAC-TCGTGGACAAGGGCACCTGCCGTG    d1
```

Osfwl1b#13, homozygote

		Genotype
Ref	TGTGCCCCTGCATCACCTTTGGGCAGGTCGCTGACATCGTGGACAAGGGCACCTGCCGTG	WT
Al1	TGTGCCCCTGCATCACCTTTGGGCAGGTCGCTGAC-TCGTGGACAAGGGCACCTGCCGTG	d1
Al2	TGTGCCCCTGCATCACCTTTGGGCAGGTCGCTGAC-TCGTGGACAAGGGCACCTGCCGTG	d1

Osfwl1b#14, bi-allele

		Genotype
Ref	TGTGCCCCTGCATCACCTTTGGGCAGGTCGCTGACA-TCGTGGACAAGGGCACCTGCCGTG	WT
Al1	TGTGCCCCTGCATCACCTTTGGGCAGGTCGCTGACAATCGTGGACAAGGGCACCTGCCGTG	i1
Al2	TGTGCCCCTGCATCACCTTTGGGCAGGTCGCTGAC--TCGTGGACAAGGGCACCTGCCGTG	d1

Osfwl2a#1, bi-allele

		Genotype
Ref	GACGAGCGGGGCGCTGTACGCGCTGGTGATGCTCCT-CACGGGCTGCAACTGCGTCTACTC	WT
Al1	GACGAGCGGGGCGCTGTACGCGCTGGTGATGCTCCTTCACGGGCTGCAACTGCGTCTACTC	i1
Al2	GACGAGCGGGGCGCTGTACGCGCTGGTGATG------CACGGGCTGCAACTGCGTCTACTC	d5

Osfwl2a#2, bi-allele

		Genotype
Ref	GACGAGCGGGGCGCTGTACGCGCTGGTGATGCTCCT-CACGGGCTGCAACTGCGTCTACTC	WT
Al1	GACGAGCGGGGCGCTGTACGCGCTGGTGATGCTCCTTCACGGGCTGCAACTGCGTCTACTC	i1
Al2	GACGAGCGGGGCGCTGTACGCGCTGGTGATGCTCC--CACGGGCTGCAACTGCGTCTACTC	d1

Osfwl2a#3, homozygote

		Genotype
Ref	GACGAGCGGGGCGCTGTACGCGCTGGTGATGCTCCT-CACGGGCTGCAACTGCGTCTACTC	WT
Al1	GACGAGCGGGGCGCTGTACGCGCTGGTGATGCTCCTTCACGGGCTGCAACTGCGTCTACTC	i1
Al2	GACGAGCGGGGCGCTGTACGCGCTGGTGATGCTCCTTCACGGGCTGCAACTGCGTCTACTC	i1

Osfwl2a#4, bi-allele

		Genotype
Ref	GACGAGCGGGGCGCTGTACGCGCTGGTGATGCTCCT-CACGGGCTGCAACTGCGTCTACTC	WT
Al1	GACGAGCGGGGCGCTGTACGCGCTGGTGATGCTCCTACACGGGCTGCAACTGCGTCTACTC	i1
Al2	GACGAGCGGGGCGCTGTACGCGC------------------GGCTGCAACTGCGTCTACTC	d17

Osfwl2a#5, bi-allele

Genotype

```
Ref    GACGAGCGGGGCGCTGTACGCGCTGGTGATGCTCCT-CACGGGCTGCAACTGCGTCTACTC    WT
Al1    GACGAGCGGGGCGCTGTACGCGCTGGTGATGCTCCTACACGGGCTGCAACTGCGTCTACTC    i1
Al2    GACGAGCGGGGCGCTGTACGCGC-----------------GGCTGCAACTGCGTCTACTC    d17
```

Osfwl2a#6, bi-allele

```
                                                                     Genotype
Ref    GACGAGCGGGGCGCTGTACGCGCTGGTGATGCTCCT-CACGGGCTGCAACTGCGTCTACTC    WT
Al1    GACGAGCGGGGCGCTGTACGCGCTGGTGATGCTCCTACACGGGCTGCAACTGCGTCTACTC    i1a
Al2    GACGAGCGGGGCGCTGTACGCGCTGGTGATGCTCCTTCACGGGCTGCAACTGCGTCTACTC    i1b
```

Osfwl2a#7, bi-allele

```
                                                                     Genotype
Ref    GACGAGCGGGGCGCTGTACGCGCTGGTGATGCTCCT--CACGGGCTGCAACTGCGTCTACTC    WT
Al1    GACGAGCGGGGCGCTGTACGCGCTGGTGATGCTCCTCCCACGGGCTGCAACTGCGTCTACTC    i2
Al2    GACGAGCGGGGCGCTGTACGCGCTGGTGATGC------CACGGGCTGCAACTGCGTCTACTC    d4
```

Osfwl2a#8, bi-allele

```
                                                                     Genotype
Ref    GACGAGCGGGGCGCTGTACGCGCTGGTGATGCTCCTCACGGGCTGCAACTGCGTCTACTC    WT
Al1    GACGAGCGGGGCGCTGTACGCGCTGGTG-------TCACGGGCTGCAACTGCGTCTACTC    d7
Al2    GACGAGCGGGGCGCTGTACGCG--------------CACGGGCTGCAACTGCGTCTACTC    d14
```

Osfwl2a#9, bi-allele

```
                                                                     Genotype
Ref    GACGAGCGGGGCGCTGTACGCGCTGGTGATGCTCCTCACGGGCTGCAACTGCGTCTACTC    WT
Al1    GACGAGCGGGGCGCTGTACGCGCTGGTGATGCTCC-CACGGGCTGCAACTGCGTCTACTC    d1
Al2    GACGAGCGGGGCGCTGTACGCGCTGGTGA-------CACGGGCTGCAACTGCGTCTACTC    d7
```

Osfwl2a#10, bi-allele

```
                                                                     Genotype
Ref    GACGAGCGGGGCGCTGTACGCGCTGGTGATGCTCCT-CACGGGCTGCAACTGCGTCTACTC    WT
Al1    GACGAGCGGGGCGCTGTACGCGCTGGTGATGCTCCTTCACGGGCTGCAACTGCGTCTACTC    i1
Al2    GACGAGCGGGGCGCTGTACGCGCTGGTGATGCTCC--CACGGGCTGCAACTGCGTCTACTC    d1
```

Osfwl2a#11, bi-allele

```
                                                                     Genotype
Ref    GACGAGCGGGGCGCTGTACGCGCTGGTGATGCTCCTCACGGGCTGCAACTGCGTCTACTC    WT
Al1    GACGAGCGGGGCGCTGTACGCGCTGGTGATG-----CACGGGCTGCAACTGCGTCTACTC    d5
```

Al2 GACGAGCGGGGCGCTGTA------------------CACGGGCTGCAACTGCGTCTACTC d18

Osfwl2a#13, bi-allele

		Genotype
Ref	GACGAGCGGGGCGCTGTACGCGCTGGTGATGCTCCTCACGGGCTGCAACTGCGTCTACTC	WT
Al1	GACGAGCGGGGCGCTGTACGCGCTGGTGATGCT---CACGGGCTGCAACTGCGTCTACTC	d3
Al2	GACGAGCGGGGCGCTGTACGCGCTGGTGATG------ACGGGCTGCAACTGCGTCTACTC	d6

Osfwl2a#14, bi-allele

		Genotype
Ref	GACGAGCGGGGCGCTGTACGCGCTGGTGATGCTCCTCACGGGCTGCAACTGCGTCTACTC	WT
Al1	GACGAGCGGGGCGCTGTACGCGCTGGTGATGCTCCT-ACGGGCTGCAACTGCGTCTACTC	d1
Al2	GACGAGCGGGGCGCTG--------------------CACGGGCTGCAACTGCGTCTACTC	d20

Osfwl2a#15, bi-allele

		Genotype
Ref	GACGAGCGGGGCGCTGTACGCGCTGGTGATGCTCCTCACGGGCTGCAACTGCGTCTACTC	WT
Al1	GACGAGCGGGGCGCTGTACGCGCTGGTGATGCTCCT-ACGGGCTGCAACTGCGTCTACTC	d1
Al2	GACGAGCGGGGCGCTG--------------------CACGGGCTGCAACTGCGTCTACTC	d20

Osfwl2a#16, bi-allele

		Genotype
Ref	GACGAGCGGGGCGCTGTACGCGCTGGTGATGCTCCTCACGGGCTGCAACTGCGTCTACTC	WT
Al1	GACGAGCGGGGCGCTGTACGCGCTGGTGATGCTC--CACGGGCTGCAACTGCGTCTACTC	d2
Al2	GACGAGCGGGGCGCTGTACGCGCTGGTGATGCT---CACGGGCTGCAACTGCGTCTACTC	d3

Osfwl2b#1, bi-allele

		Genotype
Ref	GGCTGCAACTGCGTCTACTCCTGCTTCTACCGCGCCAAGATGCGGTCCCAGTACGGCCTG	WT
Al1	GGCTGCAACTGCGTCTACTCCTGCT--TACCGCGCCAAGATGCGGTCCCAGTACGGCCTG	d2
Al2	GGCTGCAACTGCGTCTACTCCTGCT---ACCGCGCCAAGATGCGGTCCCAGTACGGCCTG	d3

Osfwl2b#2, homozygote

		Genotype
Ref	GGCTGCAACTGCGTCTACTCCTGCTTCTACCGCGCCAAGATGCGGTCCCAGTACGGCCTG	WT
Al1	GGCTGCAACTGCGTCTACTCCTGCT--TACCGCGCCAAGATGCGGTCCCAGTACGGCCTG	d2
Al2	GGCTGCAACTGCGTCTACTCCTGCT--TACCGCGCCAAGATGCGGTCCCAGTACGGCCTG	d2

Osfwl2b#3, bi-allele

		Genotype
Ref	GGCTGCAACTGCGTCTACTCCTGCTTCTACCGCGCCAAGATGCGGTCCCAGTACGGCCTG	WT
Al1	GGCTGCAACTGCGTCTACTCCTGCT-CTACCGCGCCAAGATGCGGTCCCAGTACGGCCTG	d1
Al2	GGCTGCAACTGCGTCTACTCCTGCT--TACCGCGCCAAGATGCGGTCCCAGTACGGCCTG	d2

Osfwl2b#5, homozygote

		Genotype
Ref	GGCTGCAACTGCGTCTACTCCTGCTTCTACCGCGCCAAGATGCGGTCCCAGTACGGCCTG	WT
Al1	GGCTGCAACTGCGTCTACTCCTGCT-CTACCGCGCCAAGATGCGGTCCCAGTACGGCCTG	d1
Al2	GGCTGCAACTGCGTCTACTCCTGCT-CTACCGCGCCAAGATGCGGTCCCAGTACGGCCTG	d1

Osfwl2b#7, bi-allele

		Genotype
Ref	GGCTGCAACTGCGTCTACTCCTGCT-TCTACCGCGCCAAGATGCGGTCCCAGTACGGCCTG	WT
Al1	GGCTGCAACTGCGTCTACTCCTGCTATCTACCGCGCCAAGATGCGGTCCCAGTACGGCCTG	i1
Al2	GGCTGCAACTGCGTCTACTCCTGCT---TACCGCGCCAAGATGCGGTCCCAGTACGGCCTG	d2

Osfwl2b#9, bi-allele

		Genotype
Ref	GGCTGCAACTGCGTCTACTCCTGCTTCTACCGCGCCAAGATGCGGTCCCAGTACGGCCTG	WT
Al1	GGCTGCAACTGCGTCTACTCCTGCT-CTACCGCGCCAAGATGCGGTCCCAGTACGGCCTG	d1
Al2	GGCTGCAACTGCGTCTACTCCTGCT---ACCGCGCCAAGATGCGGTCCCAGTACGGCCTG	d3

Osfwl2b#10, bi-allele

		Genotype
Ref	GGCTGCAACTGCGTCTACTCCTGCTTCTACCGCGCCAAGATGCGGTCCCAGTACGGCCTG	WT
Al1	GGCTGCAACTGCGTCTACTCCTGCT-CTACCGCGCCAAGATGCGGTCCCAGTACGGCCTG	d1
Al2	GGCTGCAACTGCGTCTACTCCTGCT-CT--CGCGCCAAGATGCGGTCCCAGTACGGCCTG	d1d2

Osfwl2b#11, bi-allele

		Genotype
Ref	GGCTGCAACTGCGTCTACTCCTGCT-TCTACCGCGCCAAGATGCGGTCCCAGTACGGCCTG	WT
Al1	GGCTGCAACTGCGTCTACTCCTGCTATCTACCGCGCCAAGATGCGGTCCCAGTACGGCCTG	i1
Al2	GGCTGCAACTGCGTCTACTCCTGCT----ACCGCGCCAAGATGCGGTCCCAGTACGGCCTG	d3

Osfwl2b#12, bi-allele

Genotype

```
Ref     GGCTGCAACTGCGTCTACTCCTGCTTCTACCGCGCCAAGATGCGGTCCCAGTACGGCCTG   WT
Al1     GGCTGCAACTGCGTCTACTCCTGCT-CTACCGCGCCAAGATGCGGTCCCAGTACGGCCTG   d1
Al2     GGCTGCAACTGCGTCTACTCCTGCT-----CGCGCCAAGATGCGGTCCCAGTACGGCCTG   d5
```

Osfwl2b#13, homozygote

```
                                                                       Genotype
Ref     GGCTGCAACTGCGTCTACTCCTGCTTCTACCGCGCCAAGATGCGGTCCCAGTACGGCCTG   WT
Al1     GGCTGCAACTGCGTCTACTCCTGCT---ACCGCGCCAAGATGCGGTCCCAGTACGGCCTG   d3
Al2     GGCTGCAACTGCGTCTACTCCTGCT---ACCGCGCCAAGATGCGGTCCCAGTACGGCCTG   d3
```

Osfwl2b#14, bi-allele

```
                                                                        Genotype
Ref     GGCTGCAACTGCGTCTACTCCTGCT-TCTACCGCGCCAAGATGCGGTCCCAGTACGGCCTG   WT
Al1     GGCTGCAACTGCGTCTACTCCTGCTTTCTACCGCGCCAAGATGCGGTCCCAGTACGGCCTG   i1
Al2     GGCTGCAACTGCGTCTACTCCTGCT---TACCGCGCCAAGATGCGGTCCCAGTACGGCCTG   d2
```

Osfwl3a#2, chimera

```
                                                                       Genotype
Ref     GTGCATCACGTTCGGGCAGATCGCGGAGATCGTCGA-CCGGGGGTCGTCGTCGTGCGGGA   WT
Al1     GTGCATCACGTTCGGGCAGATCGCGGAGATCGTCGATCCGGGGGTCGTCGTCGTGCGGGA   i1(5/10)
Al2     GTGCATCACGTTCGGGCAGATCGCGGAGATCGTCGA--CGGGGGTCGTCGTCGTGCGGGA   d1(3/10)
Al3     GTGCATCACGTTCGGGCAGATCGCGGAGAT-------CCGGGGGTCGTCGTCGTGCGGGA   d6(1/10)
Al4     GTGCATCACGTTCGGGCAGATCGCGGAGATCGTCT--CCGGGGGTCGTCGTCGTGCGGGA   d1s1(1/10)
```

Osfwl3a#3, bi-allele

```
                                                                       Genotype
Ref     GTGCATCACGTTCGGGCAGATCGCGGAGATCGTCGA-CCGGGGGTCGTCGTCGTGCGGGA   WT
Al1     GTGCATCACGTTCGGGCAGATCGCGGAGATCGTCGAACCGGGGGTCGTCGTCGTGCGGGA   i1
Al2     GTGCATCACGTTCGGGCAGATCGCGGAGATCGT----CCGGGGGTCGTCGTCGTGCGGGA   d3
```

Osfwl3a#4, chimera

```
                                                                       Genotype
Ref     TGCATCACGTTCGGGCAGATCGCGGAGATCGTCGA--CCGGGGGTCGTCGTCGTGCGGGA   WT
Al1     TGCATCACGTTCGGGCAGATCGCGGAGATCGTCGAA-CCGGGGGTCGTCGTCGTGCGGGA   i1(17/20)
Al2     TGCATCACGTTCGGGCAGATCGCGGAGATCGTCGAAACCGGGGGTCGTCGTCGTGCGGGA   i2(1/20)
Al3     TGCATCACGTTCGGGCAGATCGCGGAGA---TCGA--CCGGGGGTCGTCGTCGTGCGGGA   d3(1/20)
Al4     TGCATCACGTTCGGGCAGATCGCGGAGATCGTA-A--CCGGGGGTCGTCGTCGTGCGGGA   d1s1(1/20)
```

Osfwl3a#5, bi-allele

		Genotype
Ref	GTGCATCACGTTCGGGCAGATCGCGGAGATCGTCGA-CCGGGGGTCGTCGTCGTGCGGGA	WT
Al1	GTGCATCACGTTCGGGCAGATCGCGGAGATCGTCGAACCGGGGGTCGTCGTCGTGCGGGA	i1a
Al2	GTGCATCACGTTCGGGCAGATCGCGGAGATCGTCGATCCGGGGGTCGTCGTCGTGCGGGA	i1b

Osfwl3a#6, bi-allele

		Genotype
Ref	GTGCATCACGTTCGGGCAGATCGCGGAGATCGTCGA-CCGGGGGTCGTCGTCGTGCGGGA	WT
Al1	GTGCATCACGTTCGGGCAGATCGCGGAGATCGTCGAACCGGGGGTCGTCGTCGTGCGGGA	i1
Al2	GTGCATCACGTTCGGGCAGATCGCGGAGATCGTCGA---GGGGGTCGTCGTCGTGCGGGA	d2

Osfwl3a#7, bi-allele

		Genotype
Ref	GTGCATCACGTTCGGGCAGATCGCGGAGATCGTCGACCGGGGGTCGTCGTCGTGCGGGA	WT
Al1	GTGCATCACGTTCGGGCAGATCGCGGAGATCGTCG-CCGGGGGTCGTCGTCGTGCGGGA	d1
Al2	GTGCATCACGTTCGGGCAGATCGCGGAGATCGTC--CCGGGGGTCGTCGTCGTGCGGGA	d2

Osfwl3b#1, bi-allele

		Genotype
Ref	TACGGCCTGCAGGAGACGCCCTGCC-CCGACTGCCTCGTCCACTTGTGGTGCGAGCCCTGC	WT
Al1	TACGGCCTGCAGGAGACGCCCTGCCACCGACTGCCTCGTCCACTTGTGGTGCGAGCCCTGC	i1
Al2	TACGGCCTGCAGGAGACGCCCTGCC-C-----GCCTCGTCCACTTGTGGTGCGAGCCCTGC	d5

Osfwl3b#4, homozygote

		Genotype
Ref	TACGGCCTGCAGGAGACGCCCTGCC-CCGACTGCCTCGTCCACTTGTGGTGCGAGCCCTGC	WT
Al1	TACGGCCTGCAGGAGACGCCCTGCCACCGACTGCCTCGTCCACTTGTGGTGCGAGCCCTGC	i1
Al2	TACGGCCTGCAGGAGACGCCCTGCCACCGACTGCCTCGTCCACTTGTGGTGCGAGCCCTGC	i1

Osfwl3b#5, bi-allele

		Genotype
Ref	TACGGCCTGCAGGAGACGCCCTGCC--CCGACTGCCTCGTCCACTTGTGGTGCGAGCCCTGC	WT
Al1	TACGGCCTGCAGGAGACGCCCTGCCCCCCGACTGCCTCGTCCACTTGTGGTGCGAGCCCTGC	i2
Al2	TACGGCCTGCAGGAGACGCCCTGCC-------TGCCTCGTCCACTTGTGGTGCGAGCCCTGC	d5

Osfwl3b#6, bi-allele

Genotype

```
Ref    TACGGCCTGCAGGAGACGCCCTGCCCCGACTGCCTCGTCCACTTGTGGTGCGAGCCCTGC   WT
Al1    TACGGCCTGCAGGAGACGCCCTGCCCC----GCCTCGTCCACTTGTGGTGCGAGCCCTGC   d4
Al2    TACGGCCTGCAGGAGACGCCCTGCC-----TGCCTCGTCCACTTGTGGTGCGAGCCCTGC   d5
```

Osfwl3b#7, bi-allele

```
                                                                    Genotype
Ref    TACGGCCTGCAGGAGACGCCCTGCC--CCGACTGCCTCGTCCACTTGTGGTGCGAGCCCTGC   WT
Al1    TACGGCCTGCAGGAGACGCCCTGCCCCCCGACTGCCTCGTCCACTTGTGGTGCGAGCCCTGC   i2
Al2    TACGGCCTGCAGGAGACGCCCTGCC-------TGCCTCGTCCACTTGTGGTGCGAGCCCTGC   d5
```

Osfwl3b#8, bi-allele

```
                                                                    Genotype
Ref    TACGGCCTGCAGGAGACGCCCTGCC--CCGACTGCCTCGTCCACTTGTGGTGCGAGCCCTGC   WT
Al1    TACGGCCTGCAGGAGACGCCCTGCCCCCCGACTGCCTCGTCCACTTGTGGTGCGAGCCCTGC   i2
Al2    TACGGCCTGCAGGAGACGCCCTGCC-------TGCCTCGTCCACTTGTGGTGCGAGCCCTGC   d5
```

Osfwl3b#9, bi-allele

```
                                                                    Genotype
Ref    TACGGCCTGCAGGAGACGCCCTGCC--CCGACTGCCTCGTCCACTTGTGGTGCGAGCCCTGC   WT
Al1    TACGGCCTGCAGGAGACGCCCTGCC-ACCGACTGCCTCGTCCACTTGTGGTGCGAGCCCTGC   i1
Al2    TACGGCCTGCAGGAGACGCCCTGCCAACCGACTGCCTCGTCCACTTGTGGTGCGAGCCCTGC   i2
```

Osfwl3b#11, homozygote

```
                                                                    Genotype
Ref    TACGGCCTGCAGGAGACGCCCTGCC-CCGACTGCCTCGTCCACTTGTGGTGCGAGCCCTGC   WT
Al1    TACGGCCTGCAGGAGACGCCCTGCCACCGACTGCCTCGTCCACTTGTGGTGCGAGCCCTGC   i1
Al2    TACGGCCTGCAGGAGACGCCCTGCCACCGACTGCCTCGTCCACTTGTGGTGCGAGCCCTGC   i1
```

Osfwl3b#12, heterozygote

```
                                                                    Genotype
Ref    TACGGCCTGCAGGAGACGCCCTGCC-CCGACTGCCTCGTCCACTTGTGGTGCGAGCCCTGC   WT
Al1    TACGGCCTGCAGGAGACGCCCTGCC-CCGACTGCCTCGTCCACTTGTGGTGCGAGCCCTGC   WT
Al2    TACGGCCTGCAGGAGACGCCCTGCCACCGACTGCCTCGTCCACTTGTGGTGCGAGCCCTGC   i1
```

Osfwl3b#13, bi-allele

```
                                                                    Genotype
Ref    TACGGCCTGCAGGAGACGCCCTGCC--CCGACTGCCTCGTCCACTTGTGGTGCGAGCCCTGC   WT
Al1    TACGGCCTGCAGGAGACGCCCTGCCAACCGACTGCCTCGTCCACTTGTGGTGCGAGCCCTGC   i2a
```

Al2 TACGGCCTGCAGGAGACGCCCTGCCCCCCGACTGCCTCGTCCACTTGTGGTGCGAGCCCTGC i2b

Osfwl3b#14, bi-allele

		Genotype
Ref	TACGGCCTGCAGGAGACGCCCTGCC--CCGACTGCCTCGTCCACTTGTGGTGCGAGCCCTGC	WT
Al1	TACGGCCTGCAGGAGACGCCCTGCCAACCGACTGCCTCGTCCACTTGTGGTGCGAGCCCTGC	i2a
Al2	TACGGCCTGCAGGAGACGCCCTGCCCCCCGACTGCCTCGTCCACTTGTGGTGCGAGCCCTGC	i2b

Osfwl3b#15, bi-allele

		Genotype
Ref	TACGGCCTGCAGGAGACGCCCTGCCCCGACTGCCTCGTCCACTTGTGGTGCGAGCCCTGC	WT
Al1	TACGGCCTGCAGGAGACGCCCTGCCC-----GCCTCGTCCACTTGTGGTGCGAGCCCTGC	d5a
Al2	TACGGCCTGCAGGAGACGCCCTGCC-----TGCCTCGTCCACTTGTGGTGCGAGCCCTGC	d5b

Osfwl3b#16, bi-allele

		Genotype
Ref	TACGGCCTGCAGGAGACGCCCTGCC--CCGACTGCCTCGTCCACTTGTGGTGCGAGCCCTGC	WT
Al1	TACGGCCTGCAGGAGACGCCCTGCCCCCCGACTGCCTCGTCCACTTGTGGTGCGAGCCCTGC	i2
Al2	TACGGCCTGCAGGAGACGCCCTGCC-------TGCCTCGTCCACTTGTGGTGCGAGCCCTGC	d5

Osfwl3b#17, bi-allele

		Genotype
Ref	TACGGCCTGCAGGAGACGCCCTGCC-CCGACTGCCTCGTCCACTTGTGGTGCGAGCCCTGC	WT
Al1	TACGGCCTGCAGGAGACGCCCTGCCACCGACTGCCTCGTCCACTTGTGGTGCGAGCCCTGC	i1
Al2	TACGGCCTGCAGGAGACGCCCTGCC----ACTGCCTCGTCCACTTGTGGTGCGAGCCCTGC	d3

Osfwl3b#18, chimera

		Genotype
Ref	TACGGCCTGCAGGAGACGCCCTGCC--CCGACTGCCTCGTCCACTTGTGGTGCGAGCCCT	WT
Al1	TACGGCCTGCAGGAGACGCCCTGCC-ACCGACTGCCTCGTCCACTTGTGGTGCGAGCCCT	i1a(1/10)
Al2	TACGGCCTGCAGGAGACGCCCTGCC-TCCGACTGCCTCGTCCACTTGTGGTGCGAGCCCT	i1b(5/10)
Al3	TACGGCCTGCAGGAGACGCCCTGCCCCCCGACTGCCTCGTCCACTTGTGGTGCGAGCCCT	i2(4/10)

Osfwl3b#19, chimera

		Genotype
Ref	TACGGCCTGCAGGAGACGCCCTGCC--CCGACTGCCTCGTCCACTTGTGGTGCGAGCCCT	WT
Al1	TACGGCCTGCAGGAGACGCCCTGCC-ACCGACTGCCTCGTCCACTTGTGGTGCGAGCCCT	i1a(3/10)
Al2	TACGGCCTGCAGGAGACGCCCTGCC-TCCGACTGCCTCGTCCACTTGTGGTGCGAGCCCT	i1b(2/10)

Al3	TACGGCCTGCAGGAGACGCCCTGCCCCCCGACTGCCTCGTCCACTTGTGGTGCGAGCCCT	i2(1/10)
Al4	TACGGCCTGCAGGAGACGCCCTGCC-----ACTGCCTCGTCCACTTGTGGTGCGAGCCCT	d3(4/10)

Osfwl3b#20, bi-allele

		Genotype
Ref	TACGGCCTGCAGGAGACGCCCTGCC-CCGACTGCCTCGTCCACTTGTGGTGCGAGCCCTGC	WT
Al1	TACGGCCTGCAGGAGACGCCCTGCCACCGACTGCCTCGTCCACTTGTGGTGCGAGCCCTGC	i1(4/8)
Al2	TACGGCCTGCAGGAGACGCCCT--------------CGTCCACTTGTGGTGCGAGCCCTGC	d13(4/8)

Osfwl3b#21, chimera

		Genotype
Ref	TACGGCCTGCAGGAGACGCCCTGCC--CCGACTGCCTCGTCCACTTGTGGTGCGAGCCCT	WT
Al1	TACGGCCTGCAGGAGACGCCCTGCC-ACCGACTGCCTCGTCCACTTGTGGTGCGAGCCCT	i1a(1/10)
Al2	TACGGCCTGCAGGAGACGCCCTGCC-TCCGACTGCCTCGTCCACTTGTGGTGCGAGCCCT	i1b(4/10)
Al3	TACGGCCTGCAGGAGACGCCCTGCCCCCCGACTGCCTCGTCCACTTGTGGTGCGAGCCCT	i2(3/10)
Al4	TACGGCCTGCAGGAGACGCCCTGCC---CGACTGCCTCGTCCACTTGTGGTGCGAGCCCT	d1(1/10)
Al5	TACGGCCTGCAGGAGACGCCCTGCC-------TGCCTCGTCCACTTGTGGTGCGAGCCCT	d5(1/10)

Osfwl3b#22, chimera

		Genotype
Ref	TACGGCCTGCAGGAGACGCCCTGCC--CCGACTGCCTCGTCCACTTGTGGTGCGAGCCCT	WT
Al1	TACGGCCTGCAGGAGACGCCCTGCC-TCCGACTGCCTCGTCCACTTGTGGTGCGAGCCCT	i1(6/10)
Al2	TACGGCCTGCAGGAGACGCCCTGCCCCCCGACTGCCTCGTCCACTTGTGGTGCGAGCCCT	i2(1/10)
Al3	TACGGCCTGCAGGAGACGCCCTGCC-------TGCCTCGTCCACTTGTGGTGCGAGCCCT	d5(3/10)

Osfwl3b#23, chimera

		Genotype
Ref	TACGGCCTGCAGGAGACGCCCTGCC-CCGACTGCCTCGTCCACTTGTGGTGCGAGCCCTG	WT
Al1	TACGGCCTGCAGGAGACGCCCTGCCTCCGACTGCCTCGTCCACTTGTGGTGCGAGCCCTG	i1(4/10)
Al2	TACGGCCTGCAGGAGACGCCCTGCC--CGACTGCCTCGTCCACTTGTGGTGCGAGCCCTG	d1(2/10)
Al3	TACGGCCTGCAGGAGACGCCCTGCC---GACTGCCTCGTCCACTTGTGGTGCGAGCCCTG	d2(3/10)
Al4	TACGGCCTGCAGGAGACGCCCTGCC------TTCCTCGTCCACTTGTGGTGCGAGCCCTG	d5s1(1/10)

Osfwl3b#24, chimera

		Genotype
Ref	TACGGCCTGCAGGAGACGCCCTGCC-CCGACTGCCTCGTCCACTTGTGGTGCGAGCCCTG	WT
Al1	TACGGCCTGCAGGAGACGCCCTGCCTCCGACTGCCTCGTCCACTTGTGGTGCGAGCCCTG	i1(3/10)
Al2	TACGGCCTGCAGGAGACGCCCTGCC--CGACTGCCTCGTCCACTTGTGGTGCGAGCCCTG	d1(4/10)

Al3 TACGGCCTGCAGGAGACGCCCTGCC------TGCCTCGTCCACTTGTGGTGCGAGCCCTG d5(3/10)

Osfwl3b#25, bi-allele

		Genotype
Ref	TACGGCCTGCAGGAGACGCCCTGCC-CCGACTGCCTCGTCCACTTGTGGTGCGAGCCCTG	WT
Al1	TACGGCCTGCAGGAGACGCCCTGCCACCGACTGCCTCGTCCACTTGTGGTGCGAGCCCTG	i1
Al2	TACGGCCTGCAGGAGACGCCCTGCC----ACTGCCTCGTCCACTTGTGGTGCGAGCCCTG	d3

Osfwl4a#1, bi-allele

		Genotype
Ref	CAACACAATGACTGGTCATCCGGACTCTTCGCCTGCTTCAATGACTGCGAAGTTTGTGCG	WT
Al1	CAACACAATGACTGGTCATCCGGAC--TTCGCCTGCTTCAATGACTGCGAAGTTTGTGCG	d2
Al2	CAACACAATGACTGGTCATCCGGAC--------TGCTTCAATGACTGCGAAGTTTGTGCG	d8

Osfwl4a#2, bi-allele

		Genotype
Ref	CAACACAATGACTGGTCATCCGGACTCTTCGCCTGCTTCAATGACTGCGAAGTTTGTGCG	WT
Al1	CAACACAATGACTGGTCATCCGGAC--TTCGCCTGCTTCAATGACTGCGAAGTTTGTGCG	d2
Al2	CAACACAATGACTGGTCATCCGGAC--GTCGCCTGCTTCAATGACTGCGAAGTTTGTGCG	d2s1

Osfwl4a#3, bi-allele

		Genotype
Ref	CAACACAATGACTGGTCATCCGGACTCTTCGCCTGCTTCAATGACTGCGAAGTTTGTGCG	WT
Al1	CAACACAATGACTGGTCATCCGGAC--TTCGCCTGCTTCAATGACTGCGAAGTTTGTGCG	d2
Al2	CAACACAATGACTGGTCATCCGGAC---TCGCCTGCTTCAATGACTGCGAAGTTTGTGCG	d3

Osfwl4a#4, bi-allele

		Genotype
Ref	CAACACAATGACTGGTCATCCGGACTCTTCGCCTGCTTCAATGACTGCGAAGTTTGTGCG	WT
Al1	CAACACAATGACTGGTCATCCGGAC--TTCGCCTGCTTCAATGACTGCGAAGTTTGTGCG	d2
Al2	CAACACAATGACTGGTCATCCGGAC---TCGCCTGCTTCAATGACTGCGAAGTTTGTGCG	d3

Osfwl4a#5, bi-allele

		Genotype
Ref	CAACACAATGACTGGTCATCCGGAC-TCTTCGCCTGCTTCAATGACTGCGAAGTTTGTGCG	WT
Al1	CAACACAATGACTGGTCATCCGGACATCTTCGCCTGCTTCAATGACTGCGAAGTTTGTGCG	i1
Al2	CAACACAATGACTGGTCATCCGGA----TTCGCCTGCTTCAATGACTGCGAAGTTTGTGCG	d3

Osfwl4a#6, homozygote

		Genotype
Ref	CAACACAATGACTGGTCATCCGGAC-TCTTCGCCTGCTTCAATGACTGCGAAGTTTGTGCG	WT
Al1	CAACACAATGACTGGTCATCCGGACTTCTTCGCCTGCTTCAATGACTGCGAAGTTTGTGCG	i1
Al2	CAACACAATGACTGGTCATCCGGACTTCTTCGCCTGCTTCAATGACTGCGAAGTTTGTGCG	i1

Osfwl4a#7, homozygote

		Genotype
Ref	CAACACAATGACTGGTCATCCGGACTCTTCGCCTGCTTCAATGACTGCGAAGTTTGTGCG	WT
Al1	CAACACAATGACTGGTCATCCGGAC--TTCGCCTGCTTCAATGACTGCGAAGTTTGTGCG	d2
Al2	CAACACAATGACTGGTCATCCGGAC--TTCGCCTGCTTCAATGACTGCGAAGTTTGTGCG	d2

Osfwl4a#8, bi-allele

		Genotype
Ref	GCCGCAACACAATGACTGGTCATCCGGACTCTTCGCCTGCTTCAATGACTGCGAAGTTTGTGCG	WT
Al1	GCCGCAACACAATGACTGGTCATCCGGAC--TTCGCCTGCTTCAATGACTGCGAAGTTTGTGCG	d2
Al2	----------------------------------GCCTGCTTCAATGACTGCGAAGTTTGTGCG	d34

Osfwl4a#9, bi-allele

		Genotype
Ref	CAACACAATGACTGGTCATCCGGAC-TCTTCGCCTGCTTCAATGACTGCGAAGTTTGTGCG	WT
Al1	CAACACAATGACTGGTCATCCGGACCTCTTCGCCTGCTTCAATGACTGCGAAGTTTGTGCG	i1
Al2	CAACACAAT----------------------GCCTGCTTCAATGACTGCGAAGTTTGTGCG	d21

Osfwl4a#10, bi-allele

		Genotype
Ref	CAACACAATGACTGGTCATCCGGACTCTTCGCCTGCTTCAATGACTGCGAAGTTTGTGCG	WT
Al1	CAACACAATGACTGGTCATCCGGAC--TTCGCCTGCTTCAATGACTGCGAAGTTTGTGCG	d2
Al2	CAACACAATGACTGGTCATCCGGAC---TCGCCTGCTTCAATGACTGCGAAGTTTGTGCG	d3

Osfwl4a#12, homozygote

		Genotype
Ref	CAACACAATGACTGGTCATCCGGACTCTTCGCCTGCTTCAATGACTGCGAAGTTTGTGCG	WT
Al1	CAACACAATGACTGGTCATCCGGAC---TCGCCTGCTTCAATGACTGCGAAGTTTGTGCG	d3
Al2	CAACACAATGACTGGTCATCCGGAC---TCGCCTGCTTCAATGACTGCGAAGTTTGTGCG	d3

Osfwl4b#3, bi-allele

Genotype

```
Ref    AGATGCGCGACAGCTTCCACCTCCC-CGAGGACCCATGCTGCGACTGCTGCGTCCACGCCC   WT
Al1    AGATGCGCGACAGCTTCCACCTCCCACGAGGACCCATGCTGCGACTGCTGCGTCCACGCCC   i1a
Al2    AGATGCGCGACAGCTTCCACCTCCCCCGAGGACCCATGCTGCGACTGCTGCGTCCACGCCC   i1b
```

Osfwl4b#4, bi-allele

```
                                                                      Genotype
Ref       AGATGCGCGACAGCTTCCACCTCCCCGAGGACCCATGCTGCGACTGCTGCGTCCACGCCC   WT
Al1       AGATGCGCTC---------------------------------CTGCTGCGTCCACGCCC   d33s2
Al2 (d17)-------------------------------------------CTGCTGCGTCCACGCCC   d60
```

Osfwl4b#5, homozygote

```
                                                                   Genotype
Ref    AGATGCGCGACAGCTTCCACCTCCCCGAGGACCCATGCTGCGACTGCTGCGTCCACGCCC   WT
Al1    AGATGCGCGACAGCTTCCACCTCC------ACCCATGCTGCGACTGCTGCGTCCACGCCC   d6
Al2    AGATGCGCGACAGCTTCCACCTCC------ACCCATGCTGCGACTGCTGCGTCCACGCCC   d6
```

Osfwl4b#6, bi-allele

```
                                                                   Genotype
Ref    AGATGCGCGACAGCTTCCACCTCCC-CGAGGACCCATGCTGCGACTGCTGCGTCCACGCCC   WT
Al1    AGATGCGCGACAGCTTCCACCTCCCACGAGGACCCATGCTGCGACTGCTGCGTCCACGCCC   i1a
Al2    AGATGCGCGACAGCTTCCACCTCCCTCGAGGACCCATGCTGCGACTGCTGCGTCCACGCCC   i1b
```

Osfwl4b#7, bi-allele

```
                                                                   Genotype
Ref    AGATGCGCGACAGCTTCCACCTCCCCGAGGACCCATGCTGCGACTGCTGCGTCCACGCCC   WT
Al1    AGATGCGCGACAGCTTCCACCTCCC---GGACCCATGCTGCGACTGCTGCGTCCACGCCC   d3
Al2    AGATGCGCGACAGCTTCCACCTCC------------------ACTGCTGCGTCCACGCCC   d18
```

Osfwl4b#8, bi-allele

```
                                                                   Genotype
Ref    AGATGCGCGACAGCTTCCACCTCCCCGAGGACCCATGCTGCGACTGCTGCGTCCACGCCC   WT
Al1    AGATGCGCGACAGCTTCCACCTCCC---GGACCCATGCTGCGACTGCTGCGTCCACGCCC   d3
Al2    AGATGCGCGACAGCTTCCACCTCC------------------ACTGCTGCGTCCACGCCC   d18
```

Osfwl4b#9, bi-allele

```
                                                                   Genotype
Ref    AGATGCGCGACAGCTTCCACCTCCC-CGAGGACCCATGCTGCGACTGCTGCGTCCACGCCC   WT
Al1    AGATGCGCGACAGCTTCCACCTCCCACGAGGACCCATGCTGCGACTGCTGCGTCCACGCCC   i1
```

```
Al2    AGATGCGCGACAGCTTCCACCTCCC-------CCCATGCTGCGACTGCTGCGTCCACGCCC    d6
```

Osfwl4b#12, bi-allele

```
                                                                             Genotype
Ref    AGATGCGCGACAGCTTCCACCTCCC-CGAGGACCCATGCTGCGACTGCTGCGTCCACGCCC    WT
Al1    AGATGCGCGACAGCTTCCACCTCCCACGAGGACCCATGCTGCGACTGCTGCGTCCACGCCC    i1
Al2    AGATGCGCGACAGCTTCCACCTCCC-C-----CCCATGCTGCGACTGCTGCGTCCACGCCC    d5
```

Osfwl4b#13, chimera

```
                                                                             Genotype
Ref    ATGCGCGACAGCTTCCACCTCCC--CGAGGACCCATGCTGCGACTGCTGCGTCCACGCCC    WT
Al1    ATGCGCGACAGCTTCCACCTCCC---ACGGACCCATGCTGCGACTGCTGCGTCCACGCCC    d1s2(3/10)
Al2    ATGCGCGACAGCTTCCACCTCCCAACGAGGACCCATGCTGCGACTGCTGCGTCCACGCCC    i2(2/10)
Al3    ATGCGCGACAGCTTCCACCTCCC-------ACCCATGCTGCGACTGCTGCGTCCACGCCC    d5(3/10)
Al4    ATGCGCGACAGCTTCCACCT----------------GCTGCGACTGCTGCGTCCACGCCC    d14(2/10)
```

Osfwl4b#14, bi-allele

```
                                                                             Genotype
Ref    AGATGCGCGACAGCTTCCACCTCCCCGAGGACCCATGCTGCGACTGCTGCGTCCACGCCC    WT
Al1    AGATGCGCGACAGCTTCCACCTCCC---GGACCCATGCTGCGACTGCTGCGTCCACGCCC    d3
Al2    AGATGCGCGACAGCTTCCACCTCC------------------ACTGCTGCGTCCACGCCC    d18
```

Osfwl5a#1, bi-allele

```
                                                                             Genotype
Ref    GTGCATCCCTGTCGGCCAGATCGCAGAAATCGTCGA-CAGGGGCTCATCATGTAATTGATT    WT
Al1    GTGCATCCCTGTCGGCCAGATCGCAGAAATCGTCGAACAGGGGCTCATCATGTAATTGATT    i1a
Al2    GTGCATCCCTGTCGGCCAGATCGCAGAAATCGTCGAGCAGGGGCTCATCATGTAATTGATT    i1b
```

Osfwl5a#2, bi-allele

```
                                                                             Genotype
Ref    GTGCATCCCTGTCGGCCAGATCGCAGAAATCGTCGA-CAGGGGCTCATCATGTAATTGATT    WT
Al1    GTGCATCCCTGTCGGCCAGATCGCAGAAATCGTCGAACAGGGGCTCATCATGTAATTGATT    i1a
Al2    GTGCATCCCTGTCGGCCAGATCGCAGAAATCGTCGATCAGGGGCTCATCATGTAATTGATT    i1b
```

Osfwl5a#3, bi-allele

```
                                                                             Genotype
Ref    GTGCATCCCTGTCGGCCAGATCGCAGAAATCGTCGA-CAGGGGCTCATCATGTAATTGATT    WT
Al1    GTGCATCCCTGTCGGCCAGATCGCAGAAATCGTCGAACAGGGGCTCATCATGTAATTGATT    i1a
```

Al2   GTGCATCCCTGTCGGCCAGATCGCAGAAATCGTCGACCAGGGGCTCATCATGTAATTGATT   i1b

Osfwl5a#4, bi-allele

	Sequence	Genotype
Ref	GTGCATCCCTGTCGGCCAGATCGCAGAAATCGTCGA-CAGGGGCTCATCATGTAATTGATT	WT
Al1	GTGCATCCCTGTCGGCCAGATCGCAGAAATCGTCGAACAGGGGCTCATCATGTAATTGATT	i1a
Al2	GTGCATCCCTGTCGGCCAGATCGCAGAAATCGTCGACCAGGGGCTCATCATGTAATTGATT	i1b

Osfwl5a#5, homozygote

	Sequence	Genotype
Ref	GTGCATCCCTGTCGGCCAGATCGCAGAAATCGTCGA-CAGGGGCTCATCATGTAATTGATT	WT
Al1	GTGCATCCCTGTCGGCCAGATCGCAGAAATCGTCGATCAGGGGCTCATCATGTAATTGATT	i1
Al2	GTGCATCCCTGTCGGCCAGATCGCAGAAATCGTCGATCAGGGGCTCATCATGTAATTGATT	i1

Osfwl5a#6, homozygote

	Sequence	Genotype
Ref	GTGCATCCCTGTCGGCCAGATCGCAGAAATCGTCGA-CAGGGGCTCATCATGTAATTGATT	WT
Al1	GTGCATCCCTGTCGGCCAGATCGCAGAAATCGTCGATCAGGGGCTCATCATGTAATTGATT	i1
Al2	GTGCATCCCTGTCGGCCAGATCGCAGAAATCGTCGATCAGGGGCTCATCATGTAATTGATT	i1

Osfwl5a#7, bi-allele

	Sequence	Genotype
Ref	GTGCATCCCTGTCGGCCAGATCGCAGAAATCGTCGA-CAGGGGCTCATCATGTAATTGATT	WT
Al1	GTGCATCCCTGTCGGCCAGATCGCAGAAATCGTCGAACAGGGGCTCATCATGTAATTGATT	i1a
Al2	GTGCATCCCTGTCGGCCAGATCGCAGAAATCGTCGATCAGGGGCTCATCATGTAATTGATT	i1b

Osfwl5a#8, bi-allele

	Sequence	Genotype
Ref	GTGCATCCCTGTCGGCCAGATCGCAGAAATCGTCGA-CAGGGGCTCATCATGTAATTGATT	WT
Al1	GTGCATCCCTGTCGGCCAGATCGCAGAAATCGTCGATCAGGGGCTCATCATGTAATTGATT	i1a
Al2	GTGCATCCCTGTCGGCCAGATCGCAGAAATCGTCGAGCAGGGGCTCATCATGTAATTGATT	i1b

Osfwl5a#9, bi-allele

	Sequence	Genotype
Ref	GTGCATCCCTGTCGGCCAGATCGCAGAAATCGTCGA-CAGGGGCTCATCATGTAATTGATT	WT
Al1	GTGCATCCCTGTCGGCCAGATCGCAGAAATCGTCGAACAGGGGCTCATCATGTAATTGATT	i1a
Al2	GTGCATCCCTGTCGGCCAGATCGCAGAAATCGTCGATCAGGGGCTCATCATGTAATTGATT	i1b

Osfwl5a#10, bi-allele

```
                                                                   Genotype
Ref   GTGCATCCCTGTCGGCCAGATCGCAGAAATCGTCGA-CAGGGGCTCATCATGTAATTGATT   WT
Al1   GTGCATCCCTGTCGGCCAGATCGCAGAAATCGTCGAACAGGGGCTCATCATGTAATTGATT   i1a
Al2   GTGCATCCCTGTCGGCCAGATCGCAGAAATCGTCGATCAGGGGCTCATCATGTAATTGATT   i1b
```

Osfwl5a#11, bi-allele

```
                                                                   Genotype
Ref   GTGCATCCCTGTCGGCCAGATCGCAGAAATCGTCGA-CAGGGGCTCATCATGTAATTGATT   WT
Al1   GTGCATCCCTGTCGGCCAGATCGCAGAAATCGTCGAACAGGGGCTCATCATGTAATTGATT   i1
Al2   GTGCATCCCTGTCGGCCAGATCGCAGAAATCGTC---CAGGGGCTCATCATGTAATTGATT   d2
```

Osfwl5a#12, bi-allele

```
                                                                   Genotype
Ref   GTGCATCCCTGTCGGCCAGATCGCAGAAATCGTCGA-CAGGGGCTCATCATGTAATTGATT   WT
Al1   GTGCATCCCTGTCGGCCAGATCGCAGAAATCGTCGAACAGGGGCTCATCATGTAATTGATT   i1
Al2   GTGCATCCCTGTCGGCCAGATCGCAGAAATCGTC---CAGGGGCTCATCATGTAATTGATT   d2
```

Osfwl5a#13, bi-allele

```
                                                                   Genotype
Ref   GTGCATCCCTGTCGGCCAGATCGCAGAAATCGTCGA-CAGGGGCTCATCATGTAATTGATT   WT
Al1   GTGCATCCCTGTCGGCCAGATCGCAGAAATCGTCGAACAGGGGCTCATCATGTAATTGATT   i1
Al2   GTGCATCCCTGTCGGCCAGATCGCAGAAATCGTC---CAGGGGCTCATCATGTAATTGATT   d2
```

Osfwl5a#14, bi-allele

```
                                                                   Genotype
Ref   GTGCATCCCTGTCGGCCAGATCGCAGAAATCGTCGA-CAGGGGCTCATCATGTAATTGATT   WT
Al1   GTGCATCCCTGTCGGCCAGATCGCAGAAATCGTCGAGCAGGGGCTCATCATGTAATTGATT   i1
Al2   GTGCATCCCTGTCGGCCAGATCGCAGAAATCG-----CAGGGGCTCATCATGTAATTGATT   d4
```

Osfwl5b#1, homozygote

```
                                                                   Genotype
Ref   TTCTGCTGCCAGACCTGCTCCATCG-CACAGATGCACCGTGAGCTCAAGAACCGCGGCCAC   WT
Al1   TTCTGCTGCCAGACCTGCTCCATCGGCACAGATGCACCGTGAGCTCAAGAACCGCGGCCAC   i1
Al2   TTCTGCTGCCAGACCTGCTCCATCGGCACAGATGCACCGTGAGCTCAAGAACCGCGGCCAC   i1
```

Osfwl5b#2, bi-allele

```
                                                                   Genotype
```

Ref TTCTGCTGCCAGACCTGCTCCATCG-CACAGATGCACCGTGAGCTCAAGAACCGCGGCCAC WT
Al1 TTCTGCTGCCAGACCTGCTCCATCGACACAGATGCACCGTGAGCTCAAGAACCGCGGCCAC i1a
Al2 TTCTGCTGCCAGACCTGCTCCATCGGCACAGATGCACCGTGAGCTCAAGAACCGCGGCCAC i1b

Osfwl5b#3, homozygote

Genotype

Ref TTCTGCTGCCAGACCTGCTCCATCG-CACAGATGCACCGTGAGCTCAAGAACCGCGGCCAC WT
Al1 TTCTGCTGCCAGACCTGCTCCATCGGCACAGATGCACCGTGAGCTCAAGAACCGCGGCCAC i1
Al2 TTCTGCTGCCAGACCTGCTCCATCGGCACAGATGCACCGTGAGCTCAAGAACCGCGGCCAC i1

Osfwl5b#4, homozygote

Genotype

Ref TTCTGCTGCCAGACCTGCTCCATCG-CACAGATGCACCGTGAGCTCAAGAACCGCGGCCAC WT
Al1 TTCTGCTGCCAGACCTGCTCCATCGACACAGATGCACCGTGAGCTCAAGAACCGCGGCCAC i1
Al2 TTCTGCTGCCAGACCTGCTCCATCGACACAGATGCACCGTGAGCTCAAGAACCGCGGCCAC i1

Osfwl5b#5, homozygote

Genotype

Ref TTCTGCTGCCAGACCTGCTCCATCG-CACAGATGCACCGTGAGCTCAAGAACCGCGGCCAC WT
Al1 TTCTGCTGCCAGACCTGCTCCATCGGCACAGATGCACCGTGAGCTCAAGAACCGCGGCCAC i1
Al2 TTCTGCTGCCAGACCTGCTCCATCGGCACAGATGCACCGTGAGCTCAAGAACCGCGGCCAC i1

Osfwl5b#6, homozygote

Genotype

Ref TTCTGCTGCCAGACCTGCTCCATCG-CACAGATGCACCGTGAGCTCAAGAACCGCGGCCAC WT
Al1 TTCTGCTGCCAGACCTGCTCCATCGGCACAGATGCACCGTGAGCTCAAGAACCGCGGCCAC i1
Al2 TTCTGCTGCCAGACCTGCTCCATCGGCACAGATGCACCGTGAGCTCAAGAACCGCGGCCAC i1

Osfwl5b#7, homozygote

Genotype

Ref TTCTGCTGCCAGACCTGCTCCATCG-CACAGATGCACCGTGAGCTCAAGAACCGCGGCCAC WT
Al1 TTCTGCTGCCAGACCTGCTCCATCGACACAGATGCACCGTGAGCTCAAGAACCGCGGCCAC i1
Al2 TTCTGCTGCCAGACCTGCTCCATCGACACAGATGCACCGTGAGCTCAAGAACCGCGGCCAC i1

Osfwl5b#8, bi-allele

Genotype

Ref TTCTGCTGCCAGACCTGCTCCATCG-CACAGATGCACCGTGAGCTCAAGAACCGCGGCCAC WT
Al1 TTCTGCTGCCAGACCTGCTCCATCGACACAGATGCACCGTGAGCTCAAGAACCGCGGCCAC i1a

Al2	TTCTGCTGCCAGACCTGCTCCATCGTCACAGATGCACCGTGAGCTCAAGAACCGCGGCCAC	i1b

Osfwl5b#9, bi-allele

		Genotype
Ref	TTCTGCTGCCAGACCTGCTCCATCG-CACAGATGCACCGTGAGCTCAAGAACCGCGGCCAC	WT
Al1	TTCTGCTGCCAGACCTGCTCCATCGACACAGATGCACCGTGAGCTCAAGAACCGCGGCCAC	i1a
Al2	TTCTGCTGCCAGACCTGCTCCATCGTCACAGATGCACCGTGAGCTCAAGAACCGCGGCCAC	i1b

Osfwl5b#10, bi-allele

		Genotype
Ref	TTCTGCTGCCAGACCTGCTCCATCG-CACAGATGCACCGTGAGCTCAAGAACCGCGGCCAC	WT
Al1	TTCTGCTGCCAGACCTGCTCCATCGTCACAGATGCACCGTGAGCTCAAGAACCGCGGCCAC	i1a
Al2	TTCTGCTGCCAGACCTGCTCCATCGGCACAGATGCACCGTGAGCTCAAGAACCGCGGCCAC	i1b

Osfwl5b#11, bi-allele

		Genotype
Ref	TTCTGCTGCCAGACCTGCTCCATCG-CACAGATGCACCGTGAGCTCAAGAACCGCGGCCAC	WT
Al1	TTCTGCTGCCAGACCTGCTCCATCGACACAGATGCACCGTGAGCTCAAGAACCGCGGCCAC	i1a
Al2	TTCTGCTGCCAGACCTGCTCCATCGCCACAGATGCACCGTGAGCTCAAGAACCGCGGCCAC	i1b

Osfwl5b#12, bi-allele

		Genotype
Ref	TTCTGCTGCCAGACCTGCTCCATCG-CACAGATGCACCGTGAGCTCAAGAACCGCGGCCAC	WT
Al1	TTCTGCTGCCAGACCTGCTCCATCGACACAGATGCACCGTGAGCTCAAGAACCGCGGCCAC	i1a
Al2	TTCTGCTGCCAGACCTGCTCCATCGTCACAGATGCACCGTGAGCTCAAGAACCGCGGCCAC	i1b

Osfwl5b#13, bi-allele

		Genotype
Ref	TTCTGCTGCCAGACCTGCTCCATCG-CACAGATGCACCGTGAGCTCAAGAACCGCGGCCAC	WT
Al1	TTCTGCTGCCAGACCTGCTCCATCGACACAGATGCACCGTGAGCTCAAGAACCGCGGCCAC	i1a
Al2	TTCTGCTGCCAGACCTGCTCCATCGTCACAGATGCACCGTGAGCTCAAGAACCGCGGCCAC	i1b

Osfwl5b#14, homozygote

		Genotype
Ref	TTCTGCTGCCAGACCTGCTCCATCG-CACAGATGCACCGTGAGCTCAAGAACCGCGGCCAC	WT
Al1	TTCTGCTGCCAGACCTGCTCCATCGTCACAGATGCACCGTGAGCTCAAGAACCGCGGCCAC	i1
Al2	TTCTGCTGCCAGACCTGCTCCATCGTCACAGATGCACCGTGAGCTCAAGAACCGCGGCCAC	i1

Osfwl6a#2, bi-allele

```
                                                          Genotype
Ref   GGAGATGGTGGACAGGGGCTCGACGTCGTGCGGCAC-CGGCGGCGCGCTGTACGGGCTGCT   WT
Al1   GGAGATGGTGGACAGGGGCTCGACGTCGTGCGGCACACGGCGGCGCGCTGTACGGGCTGCT   i1a
Al2   GGAGATGGTGGACAGGGGCTCGACGTCGTGCGGCACTCGGCGGCGCGCTGTACGGGCTGCT   i1b
```

Osfwl6a#8, bi-allele

```
                                                          Genotype
Ref   GGAGATGGTGGACAGGGGCTCGACGTCGTGCGGCAC-CGGCGGCGCGCTGTACGGGCTGCT   WT
Al1   GGAGATGGTGGACAGGGGCTCGACGTCGTGCGGCACTCGGCGGCGCGCTGTACGGGCTGCT   i1
Al2   GGAGATGGTGGACAGGGGCTCGACGTCGTGCGGCA--CGGCGGCGCGCTGTACGGGCTGCT   d1
```

Osfwl6a#13, homozygote

```
                                                          Genotype
Ref   GGAGATGGTGGACAGGGGCTCGACGTCGTGCGGCAC-CGGCGGCGCGCTGTACGGGCTGCT   WT
Al1   GGAGATGGTGGACAGGGGCTCGACGTCGTGCGGCACGCGGCGGCGCGCTGTACGGGCTGCT   i1
Al2   GGAGATGGTGGACAGGGGCTCGACGTCGTGCGGCACGCGGCGGCGCGCTGTACGGGCTGCT   i1
```

Osfwl6a#14, bi-allele

```
                                                             Genotype
Ref   GGAGATGGTGGACAGGGGCTCGACGTCGTGCGGCAC-CGGCGGCGCGCTGTACGGGCTGCT      WT
Al1   GGAGATGGTGGACAGGGGCTCGACGTCGTGCGGCACTCGGCGGCGCGCTGTACGGGCTGCT      i1
Al2   GGAGATGGTGGACAGGGGC------------------------------------------(d49) d90
```

Osfwl6b#1, bi-allele

```
                                                          Genotype
Ref   CTACTCCTGCACCTACCGGGGCAAGATGCGCACTCA-GTACGGGCTCGCCGAAGCCGGCTG   WT
Al1   CTACTCCTGCACCTACCGGGGCAAGATGCGCACTCAAGTACGGGCTCGCCGAAGCCGGCTG   i1
Al2   CTACTCCTGCACCTACCGGGGCAAGATGCGCA-----GTACGGGCTCGCCGAAGCCGGCTG   d4
```

Osfwl6b#2, bi-allele

```
                                                          Genotype
Ref   CTACTCCTGCACCTACCGGGGCAAGATGCGCACTCA-GTACGGGCTCGCCGAAGCCGGCTG   WT
Al1   CTACTCCTGCACCTACCGGGGCAAGATGCGCACTCAAGTACGGGCTCGCCGAAGCCGGCTG   i1
Al2   CTACTCCTGCACCTACCGGGGCAAGATGCGCA-----GTACGGGCTCGCCGAAGCCGGCTG   d4
```

Osfwl6b#3, homozygote

```
                                                          Genotype
```

```
Ref     CTACTCCTGCACCTACCGGGGCAAGATGCGCACTCA-GTACGGGCTCGCCGAAGCCGGCTG    WT
Al1     CTACTCCTGCACCTACCGGGGCAAGATGCGCACTCAAGTACGGGCTCGCCGAAGCCGGCTG    i1
Al2     CTACTCCTGCACCTACCGGGGCAAGATGCGCACTCAAGTACGGGCTCGCCGAAGCCGGCTG    i1
```

Osfwl6b#5, bi-allele

```
                                                                    Genotype
Ref     CTACTCCTGCACCTACCGGGGCAAGATGCGCACTCAGTACGGGCTCGCCGAAGCCGGCTG    WT
Al1     CTACTCCTGCACCTACCGGGGCAAGATGCGCA----GTACGGGCTCGCCGAAGCCGGCTG    d4
Al2     CTACTCCTGCACCTACCGGGGCAAGA--------------------------------TG    d32
```

Osfwl6b#6, bi-allele

```
                                                                    Genotype
Ref     CTACTCCTGCACCTACCGGGGCAAGATGCGCACTCAGTACGGGCTCGCCGAAGCCGGCT    WT
Al1     CTACTCCTGCACCTACCGGGGCAAGAT---------GTACGGGCTCGCCGAAGCCGGCT    d9(6/10)
Al2     CTACTCCTGCACCTACCGGCGCAGCCGGC--------TACGGGCTCGCCGAAGCCGGCT    d8s10(4/10)
```

Osfwl6b#7, bi-allele

```
                                                                    Genotype
Ref     CTACTCCTGCACCTACCGGGGCAAGATGCGCACTCA-GTACGGGCTCGCCGAAGCCGGCTG    WT
Al1     CTACTCCTGCACCTACCGGGGCAAGATGCGCACTCATGTACGGGCTCGCCGAAGCCGGCTG    i1
Al2     CTACTCCTGCACCTACCGGGGCAAGATGCGCA-----GTACGGGCTCGCCGAAGCCGGCTG    d4
```

Osfwl6b#8, bi-allele

```
                                                                    Genotype
Ref     CTACTCCTGCACCTACCGGGGCAAGATGCGCACTCA-GTACGGGCTCGCCGAAGCCGGCTG    WT
Al1     CTACTCCTGCACCTACCGGGGCAAGATGCGCACTCAAGTACGGGCTCGCCGAAGCCGGCTG    i1
Al2     CTACTCCTGCACCTACCGGGGCAAGATGCGCAC--A-GTACGGGCTCGCCGAAGCCGGCTG    d2
```

Osfwl6b#9, homozygote

```
                                                                    Genotype
Ref     CTACTCCTGCACCTACCGGGGCAAGATGCGCACTCA-GTACGGGCTCGCCGAAGCCGGCTG    WT
Al1     CTACTCCTGCACCTACCGGGGCAAGATGCGCACTCAAGTACGGGCTCGCCGAAGCCGGCTG    i1
Al2     CTACTCCTGCACCTACCGGGGCAAGATGCGCACTCAAGTACGGGCTCGCCGAAGCCGGCTG    i1
```

Osfwl6b#10, homozygote

```
                                                                    Genotype
Ref     CTACTCCTGCACCTACCGGGGCAAGATGCGCACTCA-GTACGGGCTCGCCGAAGCCGGCTG    WT
Al1     CTACTCCTGCACCTACCGGGGCAAGATGCGCACTCAAGTACGGGCTCGCCGAAGCCGGCTG    i1
```

Al2   CTACTCCTGCACCTACCGGGGCAAGATGCGCACTCAAGTACGGGCTCGCCGAAGCCGGCTG   i1

Osfwl6b#11, bi-allele

		Genotype
Ref	CTACTCCTGCACCTACCGGGGCAAGATGCGCACTCA-GTACGGGCTCGCCGAAGCCGGCTG	WT
Al1	CTACTCCTGCACCTACCGGGGCAAGATGCGCACTCAAGTACGGGCTCGCCGAAGCCGGCTG	i1
Al2	CTACTCCTGCACCTACCGGGGCAAGATGCGCA-----GTACGGGCTCGCCGAAGCCGGCTG	d4

Osfwl6b#12, homozygote

		Genotype
Ref	CTACTCCTGCACCTACCGGGGCAAGATGCGCACTCA-GTACGGGCTCGCCGAAGCCGGCTG	WT
Al1	CTACTCCTGCACCTACCGGGGCAAGATGCGCACTCAAGTACGGGCTCGCCGAAGCCGGCTG	i1
Al2	CTACTCCTGCACCTACCGGGGCAAGATGCGCACTCAAGTACGGGCTCGCCGAAGCCGGCTG	i1

Osfwl6b#13, bi-allele

		Genotype
Ref	CTACTCCTGCACCTACCGGGGCAAGATGCGCACTCA-GTACGGGCTCGCCGAAGCCGGCTG	WT
Al1	CTACTCCTGCACCTACCGGGGCAAGATGCGCACTCAAGTACGGGCTCGCCGAAGCCGGCTG	i1
Al2	CTACTCCTGCACCTACCGGGGCAAGATGCGCAC--A-GTACGGGCTCGCCGAAGCCGGCTG	d2

Osfwl6b#14, bi-allele

		Genotype
Ref	CTACTCCTGCACCTACCGGGGCAAGATGCGCACTCA-GTACGGGCTCGCCGAAGCCGGCTG	WT
Al1	CTACTCCTGCACCTACCGGGGCAAGATGCGCACTCAAGTACGGGCTCGCCGAAGCCGGCTG	i1
Al2	CTACTCCTGCACCTACCGGGGCAAGATGCG-------------------------------(d14)	d44

Osfwl6b#15, homozygote

		Genotype
Ref	CTACTCCTGCACCTACCGGGGCAAGATGCGCACTCAGTACGGGCTCGCCGAAGCCGGCTG	WT
Al1	CTACTCCTGCACCTACCGGGGCAAGA--CGCGCT--GTACGGGCTCGCCGAAGCCGGCTG	d2s1d2
Al2	CTACTCCTGCACCTACCGGGGCAAGA--CGCGCT--GTACGGGCTCGCCGAAGCCGGCTG	d2s1d2

Osfwl6b#16, bi-allele

		Genotype
Ref	CTACTCCTGCACCTACCGGGGCAAGATGCGCACTCA-GTACGGGCTCGCCGAAGCCGGCTG	WT
Al1	CTACTCCTGCACCTACCGGGGCAAGATGCGCACTCAAGTACGGGCTCGCCGAAGCCGGCTG	i1
Al2	CTACTCCTGCACCTACCGGGGCAAGATGCGCAC--A-GTACGGGCTCGCCGAAGCCGGCTG	d2

Osfwl7a#1, homozygote

		Genotype
Ref	GCTGCATGACGTGCTGGTGCCCGTGCATCACGTTCG-GGCGGGTGGCGGAGATGGTGGACA	WT
Al1	GCTGCATGACGTGCTGGTGCCCGTGCATCACGTTCGTGGCGGGTGGCGGAGATGGTGGACA	i1
Al2	GCTGCATGACGTGCTGGTGCCCGTGCATCACGTTCGTGGCGGGTGGCGGAGATGGTGGACA	i1

Osfwl7a#2, bi-allele

		Genotype
Ref	GCTGCATGACGTGCTGGTGCCCGTGCATCACGTTCG-GGCGGGTGGCGGAGATGGTGGACA	WT
Al1	GCTGCATGACGTGCTGGTGCCCGTGCATCACGTTCGAGGCGGGTGGCGGAGATGGTGGACA	i1a
Al2	GCTGCATGACGTGCTGGTGCCCGTGCATCACGTTCGTGGCGGGTGGCGGAGATGGTGGACA	i1b

Osfwl7a#3, bi-allele

		Genotype
Ref	GCTGCATGACGTGCTGGTGCCCGTGCATCACGTTCG-GGCGGGTGGCGGAGATGGTGGACA	WT
Al1	GCTGCATGACGTGCTGGTGCCCGTGCATCACGTTCGTGGCGGGTGGCGGAGATGGTGGACA	i1a
Al2	GCTGCATGACGTGCTGGTGCCCGTGCATCACGTTCGCGGCGGGTGGCGGAGATGGTGGACA	i1b

Osfwl7a#4, bi-allele

		Genotype
Ref	GCTGCATGACGTGCTGGTGCCCGTGCATCACGTTCG-GGCGGGTGGCGGAGATGGTGGACA	WT
Al1	GCTGCATGACGTGCTGGTGCCCGTGCATCACGTTCGGGGCGGGTGGCGGAGATGGTGGACA	i1a
Al2	GCTGCATGACGTGCTGGTGCCCGTGCATCACGTTCGCGGCGGGTGGCGGAGATGGTGGACA	i1b

Osfwl7a#5, bi-allele

		Genotype
Ref	GCTGCATGACGTGCTGGTGCCCGTGCATCACGTTCG-GGCGGGTGGCGGAGATGGTGGACA	WT
Al1	GCTGCATGACGTGCTGGTGCCCGTGCATCACGTTCGAGGCGGGTGGCGGAGATGGTGGACA	i1a
Al2	GCTGCATGACGTGCTGGTGCCCGTGCATCACGTTCGTGGCGGGTGGCGGAGATGGTGGACA	i1b

Osfwl7a#7, heterozygote

		Genotype
Ref	GCTGCATGACGTGCTGGTGCCCGTGCATCACGTTCGGGCGGGTGGCGGAGATGGTGGACA	WT
Al1	GCTGCATGACGTGCTGGTGCCCGTGCATCACGTTCGGGCGGGTGGCGGAGATGGTGGACA	WT(3/6)
Al2	GCTGCATGACGTGCTGGTGCCCGTGCATCACGTTC-GGCGGGTGGCGGAGATGGTGGACA	d1(3/6)

Osfwl7a#8, bi-allele

Genotype

Ref	GCTGCATGACGTGCTGGTGCCCGTGCATCACGTTCG-GGCGGGTGGCGGAGATGGTGGACA	WT
Al1	GCTGCATGACGTGCTGGTGCCCGTGCATCACGTTCGAGGCGGGTGGCGGAGATGGTGGACA	i1a
Al2	GCTGCATGACGTGCTGGTGCCCGTGCATCACGTTCGGGGCGGGTGGCGGAGATGGTGGACA	i1b

Osfwl7a#9, homozygote

		Genotype
Ref	GCTGCATGACGTGCTGGTGCCCGTGCATCACGTTCG-GGCGGGTGGCGGAGATGGTGGACA	WT
Al1	GCTGCATGACGTGCTGGTGCCCGTGCATCACGTTCGTGGCGGGTGGCGGAGATGGTGGACA	i1
Al2	GCTGCATGACGTGCTGGTGCCCGTGCATCACGTTCGTGGCGGGTGGCGGAGATGGTGGACA	i1

Osfwl7a#10, bi-allele

		Genotype
Ref	GCTGCATGACGTGCTGGTGCCCGTGCATCACGTTCG-GGCGGGTGGCGGAGATGGTGGACA	WT
Al1	GCTGCATGACGTGCTGGTGCCCGTGCATCACGTTCGAGGCGGGTGGCGGAGATGGTGGACA	i1a
Al2	GCTGCATGACGTGCTGGTGCCCGTGCATCACGTTCGTGGCGGGTGGCGGAGATGGTGGACA	i1b

Osfwl7a#11, bi-allele

		Genotype
Ref	GCTGCATGACGTGCTGGTGCCCGTGCATCACGTTCG-GGCGGGTGGCGGAGATGGTGGACA	WT
Al1	GCTGCATGACGTGCTGGTGCCCGTGCATCACGTTCGAGGCGGGTGGCGGAGATGGTGGACA	i1a
Al2	GCTGCATGACGTGCTGGTGCCCGTGCATCACGTTCGTGGCGGGTGGCGGAGATGGTGGACA	i1b

Osfwl7a#12, bi-allele

		Genotype
Ref	GCTGCATGACGTGCTGGTGCCCGTGCATCACGTTCG-GGCGGGTGGCGGAGATGGTGGACA	WT
Al1	GCTGCATGACGTGCTGGTGCCCGTGCATCACGTTCGTGGCGGGTGGCGGAGATGGTGGACA	i1
Al2	GCTGCATGACGTGCTGGTGCCCGTGCAT----------------GGCGGAGATGGTGGACA	d15

Osfwl7a#13, bi-allele

		Genotype
Ref	GCTGCATGACGTGCTGGTGCCCGTGCATCACGTTCG-GGCGGGTGGCGGAGATGGTGGACA	WT
Al1	GCTGCATGACGTGCTGGTGCCCGTGCATCACGTTCGAGGCGGGTGGCGGAGATGGTGGACA	i1a
Al2	GCTGCATGACGTGCTGGTGCCCGTGCATCACGTTCGCGGCGGGTGGCGGAGATGGTGGACA	i1b

Osfwl7a#14, bi-allele

		Genotype
Ref	GCTGCATGACGTGCTGGTGCCCGTGCATCACGTTCG--GGCGGGTGGCGGAGATGGTGGACA	WT
Al1	GCTGCATGACGTGCTGGTGCCCGTGCATCACGTTCGA-GGCGGGTGGCGGAGATGGTGGACA	i1(2/7)

Al2 GCTGCATGACGTGCTGGTGCCCGTGCATCACGTTCGTTGGCGGGTGGCGGAGATGGTGGACA i2(5/7)

Osfwl7b#2, bi-allele

		Genotype
Ref	GGCTGCCAGTTCGTCTACTCCTGCG-TCTACCGGGGCAAGATGCGCGCCCAGTACGGCCTC	WT
Al1	GGCTGCCAGTTCGTCTACTCCTGCGATCTACCGGGGCAAGATGCGCGCCCAGTACGGCCTC	i1
Al2	GGCTGCCAGTTCGTCTACTCCTGCG----------GCAAGATGCGCGCCCAGTACGGCCTC	d9

Osfwl7b#4, homozygote

		Genotype
Ref	GGCTGCCAGTTCGTCTACTCCTGCG-TCTACCGGGGCAAGATGCGCGCCCAGTACGGCCTC	WT
Al1	GGCTGCCAGTTCGTCTACTCCTGCGATCTACCGGGGCAAGATGCGCGCCCAGTACGGCCTC	i1
Al2	GGCTGCCAGTTCGTCTACTCCTGCGATCTACCGGGGCAAGATGCGCGCCCAGTACGGCCTC	i1

Osfwl7b#5, homozygote

		Genotype
Ref	GGCTGCCAGTTCGTCTACTCCTGCG-TCTACCGGGGCAAGATGCGCGCCCAGTACGGCCTC	WT
Al1	GGCTGCCAGTTCGTCTACTCCTGCGCTCTACCGGGGCAAGATGCGCGCCCAGTACGGCCTC	i1
Al2	GGCTGCCAGTTCGTCTACTCCTGCGCTCTACCGGGGCAAGATGCGCGCCCAGTACGGCCTC	i1

Osfwl7b#6, bi-allele

		Genotype
Ref	GGCTGCCAGTTCGTCTACTCCTGCG-TCTACCGGGGCAAGATGCGCGCCCAGTACGGCCTC	WT
Al1	GGCTGCCAGTTCGTCTACTCCTGCGATCTACCGGGGCAAGATGCGCGCCCAGTACGGCCTC	i1
Al2	GGCTGCCAG----------------TCTACCGGGGCAAGATGCGCGCCCAGTACGGCCTC	d16

Osfwl7b#8, bi-allele

		Genotype
Ref	GGCTGCCAGTTCGTCTACTCCTGCG-TCTACCGGGGCAAGATGCGCGCCCAGTACGGCCTC	WT
Al1	GGCTGCCAGTTCGTCTACTCCTGCGTTCTACCGGGGCAAGATGCGCGCCCAGTACGGCCTC	i1a
Al2	GGCTGCCAGTTCGTCTACTCCTGCGGTCTACCGGGGCAAGATGCGCGCCCAGTACGGCCTC	i1b

Osfwl7b#9, bi-allele

		Genotype
Ref	GGCTGCCAGTTCGTCTACTCCTGCG-TCTACCGGGGCAAGATGCGCGCCCAGTACGGCCTC	WT
Al1	GGCTGCCAGTTCGTCTACTCCTGCGATCTACCGGGGCAAGATGCGCGCCCAGTACGGCCTC	i1a
Al2	GGCTGCCAGTTCGTCTACTCCTGCGGTCTACCGGGGCAAGATGCGCGCCCAGTACGGCCTC	i1b

Osfwl7b#10, bi-allele

		Genotype
Ref	GGCTGCCAGTTCGTCTACTCCTGCG-TCTACCGGGGCAAGATGCGCGCCCAGTACGGCCTC	WT
Al1	GGCTGCCAGTTCGTCTACTCCTGCGATCTACCGGGGCAAGATGCGCGCCCAGTACGGCCTC	i1a
Al2	GGCTGCCAGTTCGTCTACTCCTGCGTTCTACCGGGGCAAGATGCGCGCCCAGTACGGCCTC	i1b

Osfwl7b#11, homozygote

		Genotype
Ref	GGCTGCCAGTTCGTCTACTCCTGCG-TCTACCGGGGCAAGATGCGCGCCCAGTACGGCCTC	WT
Al1	GGCTGCCAGTTCGTCTACTCCTGCGTTCTACCGGGGCAAGATGCGCGCCCAGTACGGCCTC	i1
Al2	GGCTGCCAGTTCGTCTACTCCTGCGTTCTACCGGGGCAAGATGCGCGCCCAGTACGGCCTC	i1

Osfwl7b#12, bi-allele

		Genotype
Ref	GGCTGCCAGTTCGTCTACTCCTGCG-TCTACCGGGGCAAGATGCGCGCCCAGTACGGCCTC	WT
Al1	GGCTGCCAGTTCGTCTACTCCTGCGATCTACCGGGGCAAGATGCGCGCCCAGTACGGCCTC	i1a
Al2	GGCTGCCAGTTCGTCTACTCCTGCGTTCTACCGGGGCAAGATGCGCGCCCAGTACGGCCTC	i1b

Osfwl7b#13, homozygote

		Genotype
Ref	GGCTGCCAGTTCGTCTACTCCTGCG-TCTACCGGGGCAAGATGCGCGCCCAGTACGGCCTC	WT
Al1	GGCTGCCAGTTCGTCTACTCCTGCGTTCTACCGGGGCAAGATGCGCGCCCAGTACGGCCTC	i1
Al2	GGCTGCCAGTTCGTCTACTCCTGCGTTCTACCGGGGCAAGATGCGCGCCCAGTACGGCCTC	i1

Osfwl7b#14, heterozygote

		Genotype
Ref	GGCTGCCAGTTCGTCTACTCCTGCG-TCTACCGGGGCAAGATGCGCGCCCAGTACGGCCTC	WT
Al1	GGCTGCCAGTTCGTCTACTCCTGCG-TCTACCGGGGCAAGATGCGCGCCCAGTACGGCCTC	WT
Al2	GGCTGCCAGTTCGTCTACTCCTGCGATCTACCGGGGCAAGATGCGCGCCCAGTACGGCCTC	i1

Osfwl7b#15, homozygote

		Genotype
Ref	GGCTGCCAGTTCGTCTACTCCTGCG-TCTACCGGGGCAAGATGCGCGCCCAGTACGGCCTC	WT
Al1	GGCTGCCAGTTCGTCTACTCCTGCGTTCTACCGGGGCAAGATGCGCGCCCAGTACGGCCTC	i1
Al2	GGCTGCCAGTTCGTCTACTCCTGCGTTCTACCGGGGCAAGATGCGCGCCCAGTACGGCCTC	i1

Osfwl8b#1, bi-allele

Genotype

```
Ref    GTCGCCCGCGGCTACGACCCCAAGC-TCGGATGGCACCTCAACGTCGAGCGCGGCGCCGCT   WT
Al1    GTCGCCCGCGGCTACGACCCCAAGCCTCGGATGGCACCTCAACGTCGAGCGCGGCGCCGCT   i1
Al2    GTCGCCCGCGGCTACGACCCCAAGC-TT---TGGCACCTCAACGTCGAGCGCGGCGCCGCT   d3s1
```

Osfwl8b#2, bi-allele

```
                                                                      Genotype
Ref    GTCGCCCGCGGCTACGACCCCAAGC-TCGGATGGCACCTCAACGTCGAGCGCGGCGCCGCT   WT
Al1    GTCGCCCGCGGCTACGACCCCAAGCTTCGGATGGCACCTCAACGTCGAGCGCGGCGCCGCT   i1
Al2    GTCGCCCGCGGCTACGAC---------------------AACGTCGAGCGCGGCGCCGCT   d21
```

Osfwl8b#3, bi-allele

```
                                                                      Genotype
Ref    GTCGCCCGCGGCTACGACCCCAAGC-TCGGATGGCACCTCAACGTCGAGCGCGGCGCCGCT   WT
Al1    GTCGCCCGCGGCTACGACCCCAAGCTTCGGATGGCACCTCAACGTCGAGCGCGGCGCCGCT   i1
Al2    GTCGCCCGCGGCTACGAC---------------------AACGTCGAGCGCGGCGCCGCT   d21
```

Osfwl8b#4, bi-allele

```
                                                                      Genotype
Ref    GTCGCCCGCGGCTACGACCCCAAGC-TCGGATGGCACCTCAACGTCGAGCGCGGCGCCGCT   WT
Al1    GTCGCCCGCGGCTACGACCCCAAGCGTCGGATGGCACCTCAACGTCGAGCGCGGCGCCGCT   i1
Al2    GTCGCCCGCGGCTACGACCCCAAGC-TT---TGGCACCTCAACGTCGAGCGCGGCGCCGCT   d3s1
```

Osfwl8b#5, bi-allele

```
                                                                      Genotype
Ref    GTCGCCCGCGGCTACGACCCCAAGC-TCGGATGGCACCTCAACGTCGAGCGCGGCGCCGCT   WT
Al1    GTCGCCCGCGGCTACGACCCCAAGCTTCGGATGGCACCTCAACGTCGAGCGCGGCGCCGCT   i1
Al2    GTCGCCCGCGGCTACGACCCCAAGC-TT---TGGCACCTCAACGTCGAGCGCGGCGCCGCT   d3s1
```

Osfwl8b#6, bi-allele

```
                                                                    Genotype
Ref    GTCGCCCGCGGCTACGACCCCAAGCTCGGATGGCACCTCAACGTCGAGCGCGGCGCCGCT   WT
Al1    GTCGCCCGCGGCTACGACCCCAAGC-CGGATGGCACCTCAACGTCGAGCGCGGCGCCGCT   d1
Al2    GTCGCCCGCGGCTACGACCCCAAGC--GGATGGCACCTCAACGTCGAGCGCGGCGCCGCT   d2
```

Osfwl8b#7, bi-allele

```
                                                                      Genotype
Ref    GTCGCCCGCGGCTACGACCCCAAGC-TCGGATGGCACCTCAACGTCGAGCGCGGCGCCGCT   WT
Al1    GTCGCCCGCGGCTACGACCCCAAGCTTCGGATGGCACCTCAACGTCGAGCGCGGCGCCGCT   i1
```

```
Al2    GTCGCCCGCGGCTACGAC---------------------AACGTCGAGCGCGGCGCCGCT    d21
```

Osfwl8b#8, homozygote

```
                                                                  Genotype
Ref    GTCGCCCGCGGCTACGACCCCAAGCTCGGATGGCACCTCAACGTCGAGCGCGGCGCCGCT    WT
Al1    GTCGCCCGCGGCTACGACCCCAAGC-CGGATGGCACCTCAACGTCGAGCGCGGCGCCGCT    d1
Al2    GTCGCCCGCGGCTACGACCCCAAGC-CGGATGGCACCTCAACGTCGAGCGCGGCGCCGCT    d1
```

Osfwl8b#9, homozygote

```
                                                                   Genotype
Ref    GTCGCCCGCGGCTACGACCCCAAGC-TCGGATGGCACCTCAACGTCGAGCGCGGCGCCGCT    WT
Al1    GTCGCCCGCGGCTACGACCCCAAGCTTCGGATGGCACCTCAACGTCGAGCGCGGCGCCGCT    i1
Al2    GTCGCCCGCGGCTACGACCCCAAGCTTCGGATGGCACCTCAACGTCGAGCGCGGCGCCGCT    i1
```

Osfwl8b#10, bi-allele

```
                                                                  Genotype
Ref    GTCGCCCGCGGCTACGACCCCAAGCTCGGATGGCACCTCAACGTCGAGCGCGGCGCCGCT    WT
Al1    GTCGCCCGCGGCTACGACCCCAAGC--GGATGGCACCTCAACGTCGAGCGCGGCGCCGCT    d2
Al2    GTCGCCCGCGGCTACGACCCCAAG-------GGCACCTCAACGTCGAGCGCGGCGCCGCT    d7
```

Osfwl8b#11, bi-allele

```
                                                                   Genotype
Ref    GTCGCCCGCGGCTACGACCCCAAGC-TCGGATGGCACCTCAACGTCGAGCGCGGCGCCGCT    WT
Al1    GTCGCCCGCGGCTACGACCCCAAGCATCGGATGGCACCTCAACGTCGAGCGCGGCGCCGCT    i1a
Al2    GTCGCCCGCGGCTACGACCCCAAGCTTCGGATGGCACCTCAACGTCGAGCGCGGCGCCGCT    i1b
```

Osfwl8b#12, bi-allele

```
                                                                  Genotype
Ref    GTCGCCCGCGGCTACGACCCCAAGCTCGGATGGCACCTCAACGTCGAGCGCGGCGCCGCT    WT
Al1    GTCGCCCGCGGCTACGACCCCAAGC--GGATGGCACCTCAACGTCGAGCGCGGCGCCGCT    d2
Al2    GTCGCCCGCGGCTACGACCCCAAGCT----TGGCACCTCAACGTCGAGCGCGGCGCCGCT    d4
```

Osfwl8b#13, homozygote

```
                                                                  Genotype
Ref    GTCGCCCGCGGCTACGACCCCAAGCTCGGATGGCACCTCAACGTCGAGCGCGGCGCCGCT    WT
Al1    GTCGCCCGCGGCTACGACCCCAAGCCCGGATGGCACCTCAACGTCGAGCGCGGCGCCGCT    s1
Al2    GTCGCCCGCGGCTACGACCCCAAGCCCGGATGGCACCTCAACGTCGAGCGCGGCGCCGCT    s1
```

Osfwl8b#14, bi-allele

		Genotype
Ref	GTCGCCCGCGGCTACGACCCCAAGC-TCGGATGGCACCTCAACGTCGAGCGCGGCGCCGCT	WT
Al1	GTCGCCCGCGGCTACGACCCCAAGCATCGGATGGCACCTCAACGTCGAGCGCGGCGCCGCT	i1
Al2	GTCGCCCGCGGCTACGACCCCAAGC-TT---TGGCACCTCAACGTCGAGCGCGGCGCCGCT	d3s1

Table S2. Detection of transgenes in the T_0 plants that were not mutated.

T_0 plant	*HPT*	sgRNA	*Cas9*	*Actin*
Osfwl1a#1	+	+	+	+
Osfwl1a#2	+	–	+	+
Osfwl1a#5	+	+	+	+
Osfwl1a#6	+	+	+	+
Osfwl1a#7	+	+	+	+
Osfwl1a#8	–	–	–	+
Osfwl1a#9	+	+	+	+
Osfwl1a#15	+	+	+	+
Osfwl2a#12	+	–	–	+
Osfwl2b#4	+	–	+	+
Osfwl2b#6	+	–	+	+
Osfwl2b#8	+	–	+	+
Osfwl3a#1	+	–	–	+
Osfwl3b#2	–	–	–	+
Osfwl3b#3	+	–	+	+
Osfwl3b#10	+	–	–	+
Osfwl4a#11	+	+	–	+
Osfwl4a#13	+	+	+	+
Osfwl4a#14	+	–	–	+
Osfwl4a#15	+	–	+	+
Osfwl4b#1	+	–	–	+
Osfwl4b#2	+	–	–	+
Osfwl4b#10	+	–	–	+
Osfwl4b#11	+	–	–	+
Osfwl4b#15	+	–	–	+
Osfwl6a#1	+	–	–	+
Osfwl6a#3	+	+	+	+
Osfwl6a#4	+	+	–	+
Osfwl6a#5	+	+	+	+
Osfwl6a#6	+	+	+	+
Osfwl6a#7	+	–	–	+
Osfwl6a#9	+	–	–	+
Osfwl6a#10	+	+	+	+
Osfwl6a#11	+	+	+	+
Osfwl6a#12	+	+	+	+
Osfwl6a#15	+	+	+	+

Osfwl6b#4	+	–	+	+
Osfwl7a#6	+	–	–	+
Osfwl7b#1	+	–	–	+
Osfwl7b#3	+	+	–	+
Osfwl7b#7	+	–	–	+

Table S3. sgRNA scores of rice *FWL* gene targets predicted by sgRNA scorer 2.0.

Target	Score
Osfwl1a	-0.64
Osfwl1b	0.64
Osfwl2a	-0.38
Osfwl2b	-0.17
Osfwl3a	0.92
Osfwl3b	0.44
Osfwl4a	-0.32
Osfwl4b	0.78
Osfwl5a	0.61
Osfwl5b	-0.17
Osfwl6a	1.00
Osfwl6b	0.17
Osfwl7a	1.09
Osfwl7b	-0.04
Osfwl8b	0.08

Table S4. Inheritance of CRISPR/Cas9-induced mutations in the T_1 generation.

Line	T_0 plant		T_1 plants	
	Zygosity	Genotype	No. of plants tested	Genotype
Osfwl1a#3	Bi-allele	i1ai1b	24	4i1ai1a, 11i1ai1b, 7i1bi1b, 1i1ai2, 1i1bi2
Osfwl1b#11	Homozygote	d1d1	20	20d1d1
Osfwl2a#6	Bi-allele	i1ai1b	65	18i1ai1a, 32i1ai1b, 14i1bi1b, 1i1bd18
Osfwl2b#2	Homozygote	d2d2	24	24d2d2
Osfwl3a#4	Chimera	17i1, 1i2, 1d3, 1d1s1	20	8i1i1, 12 chi
Osfwl3b#14	Bi-allele	i2ai2b	60	15i2ai2a, 30i2ai2b, 15i2bi2b
Osfwl4a#7	Homozygote	d2d2	24	24d2d2
Osfwl4b#6	Bi-allele	i1ai1b	37	8i1ai1a, 16i1ai1b, 12i1bi1b, 1i1bWT
Osfwl5a#1	Bi-allele	i1ai1b	44	10i1ai1a, 21i1ai1b, 11i1bi1b, 1i1bi1c, 1i1aWT
Osfwl5b#1	Homozygote	i1i1	24	22i1i1, 1i1d3s1, 1i1d9
Osfwl6a#13	Homozygote	i1i1	20	20i1i1
Osfwl6b#3	Homozygote	i1i1	22	22i1i1
Osfwl7a#1	Homozygote	i1i1	24	24i1i1
Osfwl7b#11	Homozygote	i1i1	30	30i1i1
Osfwl8b#8	Homozygote	d1d1	21	21d1d1

WT, wild type; i#, # of bp inserted; i#a/b/c, same number of different nucleotides inserted at the same site; d#, # of bp deleted; s#, # of bp substituted; chi, chimera.

Table S5. Sequencing results of chimeric T_1 plants of line Osfwl3a#4.

Plant	Genotype
1	2i1, 1i2, 1d1, 2d5, 2d30, 1s2, 1d3s1
2	2i1a, 1i1b, 1i2, 1d4, 1d5a, 1d5b, 1d13, 1d20, 1d2s1
3	3i1, 1i2a, 1i2b, 1d2, 3d14, 1i1s1
4	3i1, 1d1, 2d3, 1d5, 1d13, 1d38, 1d7s1
5	4i1, 2d1, 2d2, 1d5, 1d4s1
6	6i1, 1i2, 1d1, 1d3, 1d1s1
7	3i1, 1i2a, 1i2b, 1d2, 1d5, 1d7, 1d2s1
8	5i1, 1d1, 1d6, 1d7, 1d36, 1d2s1
9	1i1, 2i2, 2d5, 1d6, 1d11, 1d14, 1d30, 1s1
10	3i1a, 6i1b, 1d8
11	3i1, 1d1, 1d3, 1d4, 1d6, 1d18, 1d30, 1d2s3
12	1i1, 5i2, 1d3, 1d5a, 1d5b, 1d8

i#, # of bp inserted; i#a/b, same number of different nucleotides inserted in the same site; d#, # of bp deleted; d#a/b, deletion of the same number of nucleotides at different sites; s#, # of bp substituted.

Table S6. Inheritance of mutations of transgene-free T_1 lines in the T_2 generation.

Line	T_1 plant		T_2 plants	
	Zygosity	Genotype	No. of plants tested	Genotype
Osfwl1a#4	Homozygote	i1i1	22	22i1i1
Osfwl1b#11	Homozygote	d1d1	22	22d1d1
Osfwl2a#6	Homozygote	i1i1	22	22i1i1
Osfwl3b#13	Homozygote	i2i2	21	21i2i2
Osfwl4a#7	Homozygote	d2d2	22	22d2d2
Osfwl4b#6	Bi-allele	i1ai1b	41	9i1ai1a, 19i1ai1b, 13i1bi1b
Osfwl6b#3	Homozygote	i1i1	20	20i1i1
Osfwl7b#11	Homozygote	i1i1	22	22i1i1

i#, number of bp inserted; i1a/b, a different nucleotide inserted in the same site; d#, number of bp deleted.

Table S7. Positively correlated genes of *OsFWL4* identified by Genevestigator.

Gene locus	Score	Annotation
LOC_Os04g06090	1	cysteine-rich receptor-like protein kinase 37 precursor, putative, expressed
LOC_Os07g23690	0.99	root cap protein 1, putative, expressed
LOC_Os11g10610	0.99	NBS-LRR disease resistance protein, putative, expressed
LOC_Os10g31250	0.99	expressed protein
LOC_Os12g36350	0.99	expressed protein
LOC_Os01g66900	0.99	expressed protein
LOC_Os08g26830	0.99	expressed protein
LOC_Os04g28360	0.99	expressed protein
LOC_Os03g63600	0.99	tetraspanin family protein, putative, expressed
LOC_Os10g39310	0.99	aspartic proteinase nepenthesin precursor, putative, expressed
LOC_Os02g48750	0.99	expressed protein
LOC_Os05g33420	0.98	xyloglucanase inhibitor, putative, expressed
LOC_Os08g31070	0.98	expressed protein
LOC_Os11g04310	0.98	cytochrome P450, putative, expressed
LOC_Os04g27830	0.98	1,2-dihydroxy-3-keto-5-methylthiopentene dioxygenase protein, putative, expressed
LOC_Os12g29710	0.98	NBS-LRR disease resistance protein, putative, expressed
LOC_Os06g19360	0.98	cadmium tolerance factor, putative, expressed
LOC_Os08g07530	0.98	expressed protein
LOC_Os03g07620	0.98	expressed protein
LOC_Os10g03360	0.98	transferase family protein, putative, expressed
LOC_Os06g49260	0.98	OsWAK65 - OsWAK receptor-like protein kinase, expressed
LOC_Os04g38460	0.98	hypothetical protein
LOC_Os09g20050	0.98	expressed protein
LOC_Os06g46080	0.98	hypothetical protein
LOC_Os12g29690	0.98	NBS-LRR disease resistance protein, putative, expressed
LOC_Os05g35820	0.97	expressed protein
LOC_Os11g11650	0.97	expressed protein
LOC_Os08g26170	0.97	retrotransposon protein, putative, unclassified
LOC_Os04g56560	0.97	proton-dependent oligopeptide transport, putative, expressed
LOC_Os08g35240	0.97	AP2 domain containing protein, expressed
LOC_Os12g25020	0.97	transposon protein, putative, CACTA, En/Spm sub-class, expressed
LOC_Os11g01118	0.97	expressed protein
LOC_Os08g15740	0.97	conserved hypothetical protein
LOC_Os12g30800	0.97	ent-kaurene synthase B, chloroplast precursor, putative
LOC_Os12g04550	0.97	expressed protein

LOC_Os12g21840	0.97	expressed protein
LOC_Os11g39730	0.97	NB-ARC domain containing protein, expressed
LOC_Os06g23980	0.97	transcription factor, putative, expressed
LOC_Os08g24290	0.97	transposon protein, putative, CACTA, En/Spm sub-class, expressed
LOC_Os03g49040	0.97	transposon protein, putative, CACTA, En/Spm sub-class, expressed
LOC_Os11g33430	0.97	plant protein of unknown function domain containing protein, expressed
LOC_Os11g38300	0.97	retrotransposon protein, putative, unclassified, expressed
LOC_Os10g30430	0.97	heavy metal-associated domain containing protein, expressed
LOC_Os07g28600	0.97	transposon protein, putative, CACTA, En/Spm sub-class, expressed
LOC_Os07g01790	0.97	expressed protein
LOC_Os10g18430	0.97	agmatine coumaroyltransferase, putative, expressed
LOC_Os11g43770	0.97	Leucine Rich Repeat family protein, expressed
LOC_Os01g02410	0.97	protein kinase domain containing protein, expressed
LOC_Os08g13300	0.96	transposon protein, putative, CACTA, En/Spm sub-class, expressed
LOC_Os05g45230	0.96	WRKY58, expressed

Table S8. Negatively correlated genes of *OsFWL4* identified by Genevestigator.

Gene locus	Score	Annotation
LOC_Os01g09420	-0.73	expressed protein
LOC_Os07g01890	-0.69	expressed protein
LOC_Os11g07940	-0.68	expressed protein
LOC_Os07g12770	-0.68	transmembrane amino acid transporter protein, putative, expressed
LOC_Os03g02830	-0.67	cell cycle control protein, putative, expressed
LOC_Os09g32600	-0.65	OsFBX334 - F-box domain containing protein, expressed
LOC_Os05g06630	-0.63	expressed protein
LOC_Os02g09150	-0.62	inorganic H+ pyrophosphatase, putative, expressed
LOC_Os06g04330	-0.61	expressed protein
LOC_Os03g53390	-0.61	expressed protein
LOC_Os07g01990	-0.6	expressed protein
LOC_Os03g06880	-0.59	kinase, pfkB family, putative, expressed
LOC_Os06g02500	-0.58	superoxide dismutase, chloroplast, putative, expressed
LOC_Os04g40180	-0.58	expressed protein
LOC_Os01g53000	-0.57	trehalose synthase, putative, expressed
LOC_Os07g04450	-0.56	expressed protein
LOC_Os06g04350	-0.56	expressed protein
LOC_Os02g32380	-0.55	expressed protein
LOC_Os02g57100	-0.53	expressed protein
LOC_Os01g59110	-0.53	indole-3-acetate beta-glucosyltransferase, putative, expressed
LOC_Os09g34060	-0.53	transcription factor RF2a, putative, expressed
LOC_Os07g02770	-0.53	OsFBL32 - F-box domain and LRR containing protein, expressed
LOC_Os01g09370	-0.53	ankyrin repeat domain-containing protein 28, putative, expressed
LOC_Os06g39906	-0.53	homeobox domain containing protein, expressed
LOC_Os01g45370	-0.53	radical SAM enzyme, putative, expressed
LOC_Os01g10100	-0.52	expressed protein
LOC_Os12g29990	-0.52	O-sialoglycoprotein endopeptidase, putative, expressed
LOC_Os12g13824	-0.52	expressed protein
LOC_Os08g02540	-0.52	adenylate kinase, putative, expressed
LOC_Os02g15270	-0.51	tyrosine-protein phosphatase YVH1, putative, expressed
LOC_Os11g13420	-0.51	RNA binding protein, putative, expressed
LOC_Os06g03840	-0.51	H-BTB4 - Bric-a-Brac, Tramtrack, Broad Complex BTB domain with H family conserved sequence, expressed
LOC_Os05g50420	-0.5	retrotransposon protein, putative, unclassified, expressed
LOC_Os04g38850	-0.5	expressed protein
LOC_Os03g12000	-0.5	DEAD-box ATP-dependent RNA helicase, putative, expressed

LOC_Os11g31770	-0.5	expressed protein
LOC_Os07g01910	-0.5	expressed protein
LOC_Os07g47260	-0.5	H-BTB7 - Bric-a-Brac, Tramtrack, Broad Complex BTB domain with H family conserved sequence, expressed

Table S9. Primers used in this study.

Experiment	Primer name	Sequence (5′ to 3′)
Detection of on-target mutations	Osfwl1aF	TCTTGCCTTCCGCTTTAGCA
	Osfwl1aR	GCACACGCATGTGATGAGAC
	Osfwl1bF	ATGGACGATCCCGGAAACTG
	Osfwl1bR	AGAAGCACGAGTACAGGCAC
	Osfwl2aF	CTCGATCGATCAGCGTGTCA
	Osfwl2aR	TGATTGATCGTTGTGCGTGC
	Osfwl2bF	CTCGATCGATCAGCGTGTCA
	Osfwl2bR	TGATTGATCGTTGTGCGTGC
	Osfwl3aF	TTCTTGAGCTCGCGGTACTC
	Osfwl3aR	CACCGACCTCTTTGACTGCT
	Osfwl3bF	AGCTGCAGCACAGACTCAAA
	Osfwl3bR	GCGCAGGTTTTATGACGTGG
	Osfwl4aF	CACACCGTCGTCAAGCAAC
	Osfwl4aR	TCATGCTCATCGCTCCTAGC
	Osfwl4bF	GGCCGGCAGCTAATTTGAAG
	Osfwl4bR	GTTGCTTGACGACGGTGTG
	Osfwl5aF	CACGCTGTGCAAAACGTACA
	Osfwl5aR	GGCATGTTCTACCCTGCTCC
	Osfwl5bF	TGGATGTACGATTGAGCTACTGG
	Osfwl5bR	TCGCAGAAATCGTCGACAGG
	Osfwl6aF	ATAAAGCGTCCTCCCACTC
	Osfwl6aR	CTAACAATGCTCATCGTCCT
	Osfwl6bF	ATAAAGCGTCCTCCCACTC
	Osfwl6bR	CTAACAATGCTCATCGTCCT
	Osfwl7aF	TGTTGCACCAGAAGTGGACG
	Osfwl7aR	TCTTGGACTGCTTCGACGAC
	Osfwl7bF	TGTTGCACCAGAAGTGGACG
	Osfwl7bR	TCTTGGACTGCTTCGACGAC
	Osfwl8bF	AGGAGATGGCCAAGCCAAGC
	Osfwl8bR	ACTCACTACCTCCGTCTCAA
Detection of off-target mutations	Osfwl1aOFF-1F	CTCGGCACCTGCTAACTGAT
	Osfwl1aOFF-1R	GCGACTATGGCAAGTGGTAA
	Osfwl1aOFF-2F	ATTAAGGGAAATGGGTGATAC
	Osfwl1aOFF-2R	TAAAGCCAAAGAACATACTGG
	Osfwl1bOFF-1F	TTCCACAGAACTCCATCCCAACT
	Osfwl1bOFF-1R	CCCGTCCACGTCTTCTTGC

Osfwl1bOFF-2F	TGGCGAATAAGCTCATTTC
Osfwl1bOFF-2R	ATTTCGGTCGTTGCTCATAG
Osfwl2aOFF-1F	CTCTTCCTCAATCCCTATCC
Osfwl2aOFF-1R	AATGCGGTACTGTTTCATCT
Osfwl2aOFF-2F	TGTCAATGATTACATCCAGCAC
Osfwl2aOFF-2R	AATGAATACAGTTGGGAAGAAG
Osfwl2bOFF-1F	GTATCAAACGTGGAATGAAAGC
Osfwl2bOFF-1R	TCGTGGTTATTGTGGGTGC
Osfwl2bOFF-2F	CAGCACAGACTCAAATACGCAATG
Osfwl2bOFF-2R	TAAGCAAGCCGGGCTACGAT
Osfwl3aOFF-1F	GCAGCACGCTTCAGTTCATCA
Osfwl3aOFF-1R	GGGTGACCACCTAAGTATCTCCAA
Osfwl3aOFF-2F	GGCTCGTATAGTCGTATGTAGGG
Osfwl3aOFF-2R	GTTGTGCGTGCGGCAGGTT
Osfwl3bOFF-1F	GGACAACCCACCATTGACGA
Osfwl3bOFF-1R	CCAAAGCCCATCCACTACCC
Osfwl3bOFF-2F	TCTGCTAAGCCTGGCTATGTG
Osfwl3bOFF-2R	CGGCAACTGTAAGTAGTAATCCC
Osfwl4aOFF-1F	GTTGCAGGAAACTGAAACATG
Osfwl4aOFF-1R	CTGTAGAACAATGCCAATCAC
Osfwl4aOFF-2F	GGAGCAAACACCGAACAACT
Osfwl4aOFF-2R	TTCTTCCTGGAGATCGCCTA
Osfwl4bOFF-1F	ACAAATCGACAGAAAATCCAAA
Osfwl4bOFF-1R	CTCCGTCTTTCTCGATATTCCT
Osfwl4bOFF-2F	TCCTGGTTATTAAGGGTTT
Osfwl4bOFF-2R	TCCTGGCTGTTGAGGTAGA
Osfwl5aOFF-1F	TTCATCAATCTCGGCTTCG
Osfwl5aOFF-1R	TGCCACTACCGTCTTCCCT
Osfwl5aOFF-2F	GCACCAGCAGCGTGTACCTT
Osfwl5aOFF-2R	TCCCTTGCATTGACCGTCTC
Osfwl5bOFF-1F	CGGGAGCATCACGCATCAGC
Osfwl5bOFF-1R	CGGGCAGTGCAGTAAGAATGTCTA
Osfwl5bOFF-2F	GGTACTCAACTGCCCAGAAA
Osfwl5bOFF-2R	AATGTGGATGTGGCTACGAC
Osfwl6aOFF-1F	GGTAGACGCAGGAGTAGACGA
Osfwl6aOFF-1R	ATGCTCATTCCCACCACAAG
Osfwl6aOFF-2F	GCCTTCTTCGCCACCTAC
Osfwl6aOFF-2R	CGAACACCTTCACCTGCTT

	Osfwl6bOFF-1F	GACGCTATTTAACGGCCCATG
	Osfwl6bOFF-1R	TTCCCAGCACTAGCAGCAACTC
	Osfwl6bOFF-2F	AACGACAAGGCAGCCAATGAT
	Osfwl6bOFF-2R	GGCGCAACTGATGTGCAATAC
	Osfwl7aOFF-1F	TGTCCAGCTAAATTCAACAATC
	Osfwl7aOFF-1R	TAAAGCGTCCTCCCACTC
	Osfwl7aOFF-2F	TCCATCCGCATCCTCTTT
	Osfwl7aOFF-2R	CTCACCAGGGCTTACACG
	Osfwl7bOFF-1F	CAGGCCCAAACACCCAGTT
	Osfwl7bOFF-1R	ACCCGTCGGACATCACCAT
	Osfwl7bOFF-2F	AGGGCTCGCAGAAGAAGT
	Osfwl7bOFF-2R	GCGGCAACTGTAAGTAGTAATC
	Osfwl8bOFF-1F	AAACCTGACCATGCGTCACCA
	Osfwl8bOFF-1R	TACCAGCAACTCCATCACATCCTTA
	Osfwl8bOFF-2F	GACCGTAGAATTGTTCTAATCAC
	Osfwl8bOFF-2R	AGTGTTGGGACTATTGTGGTT
Detection of transgenes	HPTF	GGGTGTCACGTTGCAAGACC
	HPTR	ATGCCTCCGCTCGAAGTAGC
	Cas9F	CACCATCTACCACCTGAGAA
	Cas9R	CGAAGTTGCTCTTGAAGTTG
	sgRNAF	TCCCAGTCACGACGTTGTAA
	sgRNAR	GGCCATTTGTCTGCAGAAT
	ActinF	CCCCTCCTGAAAGGAAGTA
	ActinR	GGTCCGAAGAATTAGAAGCA
qRT-PCR analysis	OsFWL4F	ATGACTGCGAAGTTTGTTGCT
	OsFWL4R	CCCCTCGACACGATCTCC
	Cas9RT-F	AAACAGCAGATTCGCCTGGA
	Cas9RT-R	TCATCCGCTCGATGAAGCTC
	ActinRT-F	CTTGGCATCTCTCAGCACATT
	ActinRT-R	TTGGCTTAGCATTCTTGGGT
